# Safety and glycemic outcomes of do-it-yourself AndroidAPS hybrid closed-loop system in adults with type 1 diabetes

**DOI:** 10.1371/journal.pone.0248965

**Published:** 2021-04-05

**Authors:** Andrzej Gawrecki, Dorota Zozulinska-Ziolkiewicz, Magdalena A. Michalak, Anna Adamska, Michal Michalak, Urszula Frackowiak, Justyna Flotynska, Monika Pietrzak, Szymon Czapla, Bernhard Gehr, Aleksandra Araszkiewicz

**Affiliations:** 1 Department of Internal Medicine and Diabetology, Poznan University of Medical Sciences, Poznan, Poland; 2 Department of Diabetology and Internal Medicine, Raszeja Hospital, Poznan, Poland; 3 Department of Computer Sciences and Statistics, Poznan University of Medical Sciences, Poznan, Poland; 4 Nightscout Poland, Kielce, Poland; 5 Zentrum für Diabetes und Stoffwechselerkrankungen, m&i Fachklinik, Bad Heilbrunn, Germany; Medizinische Universitat Graz, AUSTRIA

## Abstract

**Background:**

The aim of the study was to assess the safety and glycemic outcomes with the use of a Do-It-Yourself (DIY) Hybrid Closed-Loop (HCL) system based on the AndroidAPS application in type 1 diabetes (T1D).

**Methods:**

Single-center clinical trial, with 3-week run-in and 12-week study period. DIY HCL system consisted of the Dana Diabecare RS insulin pump, Dexcom G5 continuous glucose monitoring system and AndroidAPS application. Primary outcome was safety: incidences of severe hypoglycemia, diabetic ketoacidosis, time spent in glycemia <54 mg/dl. Secondary endpoints included percentage of time in range (TIR) 70–180 mg/dl, time below 70 mg/dl, HbA1c, insulin requirements, and body weight.

**Results:**

In total 12 subjects (5 men, 7 women) were enrolled, mean age 31.3±6.7, 95%CI(27.7–34.9) years, mean diabetes duration 16.1±5.7, 95%CI(13.0–19.2) years. No episodes of severe hypoglycemia or ketoacidosis were observed. Percentage of time spent in glycemia below 54mg/dl was not increased. Average sensor glycemia was lower in the study period than baseline (141.1 ± 8.4, 95%CI(136.3–145.9) vs. 153.3 ± 17.9, 95%CI(143.2–163.4), mg/dl p<0.001). TIR 70–180 mg/dl was improved by 11.3%, 95%CI(2.8%-19.8%) (from 68.0 ± 12.7 to 79.3 ± 6.4%, p<0.001), without increasing hypoglycemia time. The HbA1c level decreased by -0.5%, 95%CI(-0.9%–-0.1%) (from 6.8 ± 0.5 to 6.3 ± 0.4%, p<0.001). Additionally, in the last 4 weeks of the study period participants significantly improved and showed TIR 70–180 mg/dl 82.1 ± 5.6%, 95%CI(78.9–85.3), time <54 mg/dl 0.30 (0.20–0.55)%, median 95%CI(0.1–0.7) and <70 mg/dl 1.90 (1.10–3.05)%, median 95%CI(0.7–3.2). The insulin requirement and body weight did not change in the study.

**Conclusions:**

The study revealed safety of the Do-It-Yourself HCL system AndroidAPS in adults with T1D, limited to well-controlled, highly selected and closely monitored patients. The use of AndroidAPS significantly improved HbA1c, time in range and average sensor glycemia without increasing hypoglycemia. As both patients and their medical team are gaining experience using the system over time, they improve glycemic control.

**Trial registration:**

German Clinical Trials Register: no. DRKS00015439; https://www.drks.de/drks_web/navigate.do?navigationId=trial.HTML&TRIAL_ID=DRKS00015439.

## Introduction

Despite the implementation of traditional insulin pumps and continuous glucose monitoring (CGM) systems in the treatment of type 1 diabetes (T1D), in all age groups a substantial part of the patients do not achieve satisfactory metabolic control [1,2]. In addition, severe, as well as, mild hypoglycemia episodes have not been eliminated [3].

In the last decade, there has been an increase in the number of research projects worldwide testing Hybrid Closed-Loop (HCL) systems to improve glycemic control in patients with T1D [2,4–13]. Currently in the research phase, HCL systems are based on various mathematical algorithms and various types of continuous subcutaneous insulin infusion (CSII) and CGM systems. These systems consist of single-hormone delivery (“insulin only”) or include simultaneous administration of glucagon (dual-hormone delivery). The safety and feasibility of different systems have been studied firstly under controlled inpatient conditions and then in the outpatient in-home setting. Positive results demonstrating safety and efficacy of several HCL systems in improving time in range without increasing or even decreasing time in hypoglycemia have been described [2,4–12].

The first commercial device constituting a hybrid closed-loop system is the Medtronic MiniMed^™^ 670G system, which was approved for use by the Food and Drug Administration (FDA) in 2016 and in 2020 the MiniMed^™^ 780G received CE (Conformité Européenne) Marking and is available in several European countries. Both consist of a CGM and an insulin pump with integrated algorithm that can be used together only [14,15]. The first “stand alone” interoperable automated glycemic controller approved by the FDA is the Tandem Control IQ algorithm (2019) [7,9]. In Europe, the first available “stand alone” algorithm is “CamAPS”, commercially available in the United Kingdom since 2020. Diabeloop is another advanced commercial system, CE-marked for its DBLG1^™^ System in Europe since 2018 but not yet available [16]. A substantial number of other manufacturers are working on hybrid-closed-loop systems and algorithms in different stages of development.

Since the availability of regulatory approved systems for patients with T1D is still limited, patients have developed so called “Do-It-Yourself Artificial Pancreas Systems” (DIY APS). There are three systems, OpenAPS, Loop for iOS and AndroidAPS. They use the following pieces of technology: CGM, insulin pump, small computer (OpenAPS only), smartphone and pump communication device (“Rileylink”, Loop for iOS only). The CGM system sends glucose values via Bluetooth to the smartphone app that controls insulin delivery by using an algorithm that has been developed and tested by a community of users. The most popular of these systems is AndroidAPS (AAPS), created by Milos Kozak and many others. It is an implementation of the first DIY APS “OpenAPS“, adjusted to a smartphone app that is relatively easy to install and to use. OpenAPS works as HCL system using a model predictive control (MPC) algorithm taking into account current glucose level, insulin dose, carbohydrate consumption and personal configuration [17,18]. The system estimates the glycemia projection every 5 minutes and adjusts the basal rates. Entering the amount of carbohydrates is required. The Real-World data published by Braune et al. show Time-In-Range (TIR) of 80.7% [19]. Furthermore, there are data from a retrospective analysis in which patients self-reported improved HbA1c level and quality of life [20]. Petruzelkova et al. showed safety of Do-It-Yourself AndroidAPS HCL system in a short pilot study during a winter camp [2]. In 2020 Toffanin et al. used in silico testing (the UVA/Padova simulator) as a pre-clinical tool to assess the safety of AndroidAPS with the goal to obtain regulatory approvals for clinical studies [21]. DIY systems by definition must be self-built and as for now are not regulated or approved by FDA nor gained CE mark. It is important to note that compared to commercial HCL systems, controlled clinical trials designed to demonstrate safety and effectiveness of DIY APS are missing; accordingly, they have no regulatory body approval. Additionally, in May 2019 FDA has issued a warning against the use of unauthorized diabetes devices after one case of hypoglycemia on DIY-APS. The systems, therefore, must be used wisely, as patients are relying on unsupported software and connectivity issues. However, the number of patients using DIY APS is increasing.

The aim of the study is to assess safety and glycemic outcomes during application of a hybrid closed-loop system based on the Do-It-Yourself Artificial Pancreas System “AndroidAPS” in patients with type 1 diabetes.

## Materials and methods

### Study design

**Fig 1:**
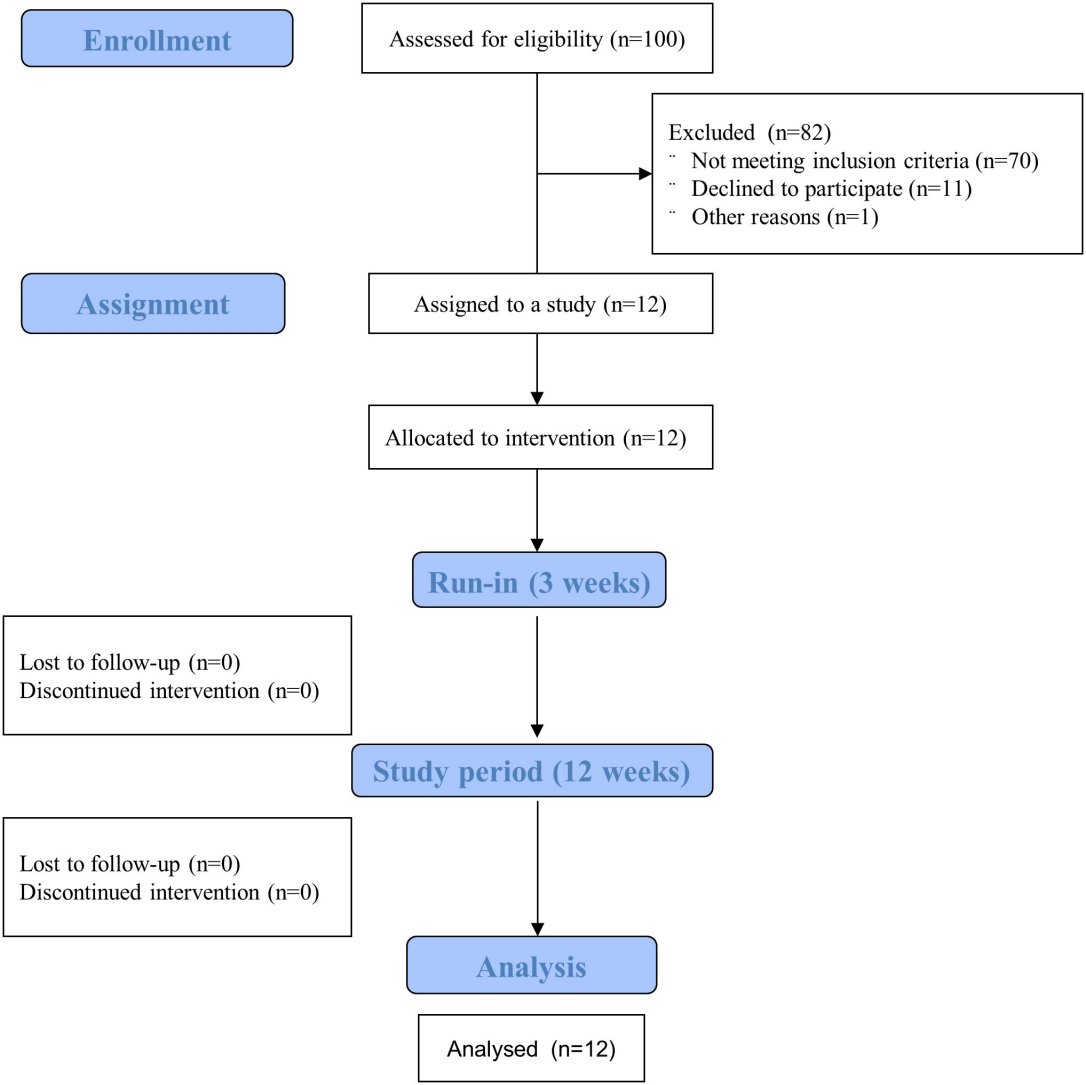
Flowchart of the patients’ recruitment.

**Fig 2:**
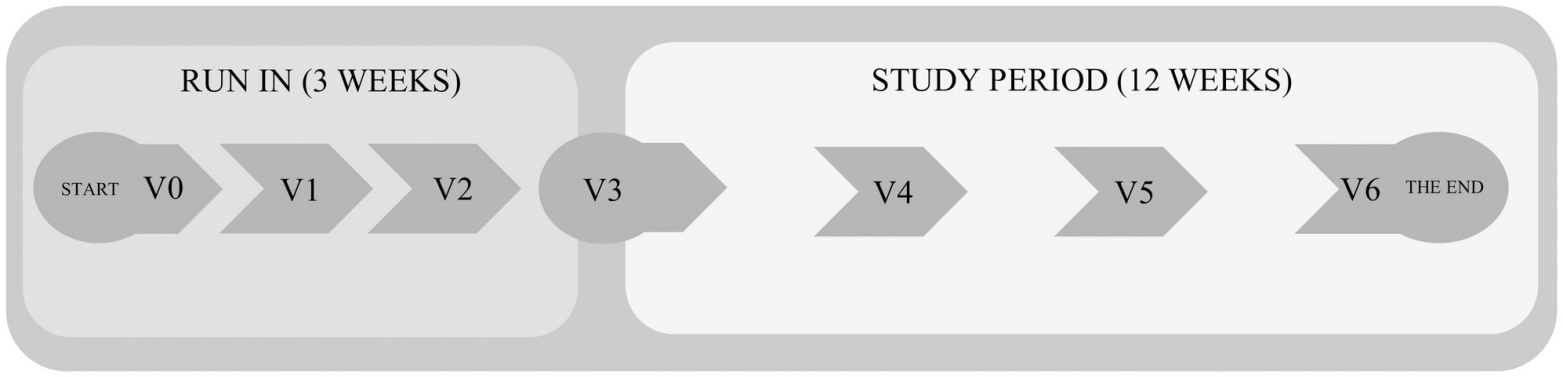
Flow diagram—Study design. Run-in visits were performed every 1 week and study period visits every 4 weeks.

This single-center, clinical trial consisted of a 3-week run-in period and a 12-week study period during real life conditions (Figs 1 and 2) and was conducted at Department of Internal Medicine and Diabetology, Poznan University of Medical Sciences. The research protocol (S1 Study Protocol in S1 File) was approved by the Bioethical Committee of Poznan University of Medical Sciences (No. 710/18, S2 File) and was registered with German Clinical trials Register: no. DRKS00015439. Written informed consent was obtained from all the participants.

### Patients

We recruited consecutive patients with type 1 diabetes who visited diabetes outpatient clinic at the Department of Internal Medicine and Diabetology at the Poznan University of Medical Sciences and met the following inclusion criteria: aged 18–45, type 1 diabetes for >5 years, treated with personal pump therapy for >1 year, using bolus calculator, with experience in CGM systems (using CGM ≥70% of the time in the last 3 months), with HbA1c <9%, BMI 18–30 kg/m^2^ and willing to participate in the research project.

The exclusion criteria were: cigarette smoking, identified advanced chronic micro- or macroangiopathic complications of diabetes (proliferative retinopathy, diabetic kidney disease with eGFR <60 mL/min/1.73 m^2^, cardiac autonomic neuropathy, coronary heart disease, stroke), severe hypoglycemia in the last year (defined as assistance by another person), hypoglycemia unawareness, diabetic ketoacidosis in the last 3 months, accompanying diseases except hypothyroidism in the state of euthyreosis, eating disorders or depression. These exclusion criteria were stated to select homogenous group of patients as we were aware of the limited number of patients available for inclusion due to obligatory CGM use (limited CGM reimbursement in Poland). We excluded smokers as we assumed that smoking could influence insulin requirement in an individual patient that could affect the results especially in a small study group [22].

### Study procedures

Enrolled patients received a DIY APS which was composed of the following elements: the Dana Diabecare RS insulin pump (SOOIL Development, Nambusunhwan-ro, Gangnam-gu, Seoul, Korea), the Dexcom G5 continuous glucose monitoring system (Dexcom Inc, San Diego, California, USA) and AndroidAPS application on Android Smartphone (Xiaomi Mi A2 Lite, Android 9). The smartphones were locked. The use of acetaminophen was forbidden. All patients used CONTOUR Plus ONE blood glucose meter (Ascensia Diabetes Care) and were requested to calibrate the sensor at least 2 times a day. All participants were gradually trained in the use of these devices during the 3-week run-in phase of the study. The participants were seen by the study staff every week during run-in. The study period consisted of a 12-week outpatient phase with intermittent control visits every 4 weeks. During the study, participants were remotely monitored in real time by the study medical team (doctors and a nurse) via the Nightscout cloud. They had immediate access to the medical personnel through phone calls and WhatsApp communicators. However, the team only intervened if patients had any questions. The data regarding glycemic outcomes and insulin doses were downloaded at scheduled visits.

#### Study procedures during study visits were as follows (Fig 1)

Visit 0 (V0)—Baseline: informed consent, data on sex, age, diabetes duration, medical history, medications, anthropometric examination, data downloading on last 4-week glycemia and insulin dose, laboratory evaluation, setting up Nightscout personal accounts, downloading AndroidAPS application, connecting the Dexcom G5 system.

Visit 1 (V1)—Connection/switch to Dana Diabecare RS pumps; patients’ preparation procedure for connecting HCL system, first steps: system visualization, analysis of the basal rate and insulin/carbohydrate ratio, open system—Temporary basal rate recommendations, Low glucose suspend function.

Visit 2 (V2)–Analysis of patient’s skills in given tasks and permission for subsequent stages of application: "tuning of the closed loop", activation of automatic change of the basal rate, subsequent correction of insulin/carbohydrate ratio and basal rate, hyperglycemia correction function using the application—correction of the basal infusion instead of correction bolus, administration of boluses only with carbohydrates intake. The patients performed all these steps under the supervision of the medical team and Nightscout representatives. The participants did not have to be specifically trained or skilled in the technical aspects of the parameters optimization of DIY system beforehand.

The run-in period (V0-V3) lasted 3 weeks and the visits were performed every week. The V0 visits lasted 3 hours, V1 visits lasted 90 to 120 minutes. The V2 visit lasted two days of hospital stay during which the closed loop system was activated.

Visit 3 (V3)–Start of the study period, technical control of HCL system and check-up of patient’s skills. Anthropometric examination, laboratory evaluation. Data on glycemia and insulin dose downloading.

Visit 4 (V4) and Visit 5 (V5) every 4 weeks—Intermittent visits in the study period. Anthropometric examination, laboratory evaluation. Data on glycemia and insulin dose downloading.

Visit 6 (V6)– 12-week end of the study period. Anthropometric examination, laboratory evaluation. Data on glycemia and insulin dose downloading.

On Visits 3–6 the primary basal rate and bolus calculator settings were modified, if indicated. However, the settings of the AndroidAPS app, on the basis of which the primary basal rate was modified, were equal for all patients and were not modified during the whole study.

Body mass index (BMI) was calculated as weight to height squared (kg/m^2^). Daily Dose of Insulin (DDI) was calculated and presented as insulin dose per patient’s body weight.

#### Laboratory procedures

Blood samples were collected in a fasting state using S-Monovette blood collection system. Glycated hemoglobin (HbA1c) was measured using competitive turbidimetric inhibition immunoassay and serum fructosamine concentration using a colorimetric method (Cobas 6000, Roche Diagnostics, Basel, Switzerland).

#### AndroidAPS hybrid closed-loop system

The application was created by two main programmers and AndroidAPS community developers: Milos Kozak and Adrian Tappe. A special version, AndroidAPS 2.0 Build: e84459c6, has been created for the purposes of the study with extended export data function. Polish representatives of NightScout also contributed to the project (technical support throughout the study, presence at V0 and at closing the loops—V2). AndroidAPS is an open source app for people living with diabetes mellitus type 1 that acts as an artificial pancreas system (APS) on Google Android smartphones. Android APS is an application based on the Affero General Public License (Affero GPL). The main application code is available at https://github.com/MilosKozak/AndroidAPS. Project documentation can be found in https://androidaps.readthedocs.io/.

The basis of the AndroidAPS application is a special algorithm that, after providing all input values, is able to estimate the future change in the basal rate needed to reach the set level by OpenAPS Advanced Meal Assist (AMA). It requires the following input for proper operation:

- current blood glucose readings, average delta of 5 minutes glycemia changes from last 5 minutes, 15 minutes and 40 minutes, these data are from CGM
- information on active insulin—Insulin On Board (IOB)
- information on the current temporary dose—temp. basal
- information about the meal, the amount of carbohydrates and insulin administrated, insulin/carbohydrate counter
- information on active carbohydrates—Carbohydrates On Board (COB)
- patient profile information: target glycemic values, insulin sensitivity factor—ISF, carbohydrate absorption profile calculated by the application based on automatic modification of the basal infusion after a meal

Based on this information, the algorithm will generate a prediction of the patient’s glucose level within the next 5 hours, taking into account different models. If the result of these calculations is different from the target range of glycemia, the deviation will be calculated. This value will be used to provide an additional correction dose of insulin or to modify the basal rate temporarily (0 up to 500 percent, depending on individual limits). Our research was based on working with xDrip+ and using its algorithm to determine glycemia. Glycemia data that was the result of the xDrip+ algorithm interpretation were immediately used for calculations in AndroidAPS. In addition, they were saved in the smartphone’s memory (Data Export Study) and exported to a database on the Heroku website. Interrupted communication with the CGM transmitter or no sensor signals resulted in switching to 100% basal rate after 30 minutes. All participants had the same version of the AndroidAPS app and the same settings according to the protocol (target glycemia level 110 mg/dl, maximal increase in the basal rates 500%, time of active insulin 5 hours, time of active carbohydrates 5 hours). These settings might be individual, but we wanted to have them equal for all the patients for the whole study duration. Five hour time of active insulin is a minimum time that is possible to set up in the system. The patients uploaded the AndroidAPS app and gradually connected all elements of the HCL system (Dana Diabecare RS, Dexcom G5) by themselves under the supervision of the medical team and IT representatives of Nightscout community. During the study no updates of the application were made.

### Statistical methods

The normality of data was tested using Shapiro-Wilks test. Categorical data are reported as numbers and percentages. Continuous variables are presented as Means and Standard Deviations (SD) or Median and Interquartile Range (IQR). To compare patients’ results between study period, run-in and baseline phase Repeated Measures Analysis of Variance was performed. The sphericity assumption was checked by Mauchly’s test. In the case of a significant ANOVA result was observed, a further post-hoc analysis (Tukey’s test) was performed in order to find homogenous groups. Outcomes with non-normal distributions were compared by Friedman test with Dunn’s post-hoc tests. The differences between categorical data were analyzed by test for proportions. A group of 12 participants was arbitrarily selected and, than *Post-hoc* analysis of "observed power" was conducted after a study has been completed. Statistical analyses were performed using TIBCO Software Inc. (2017). Statistica (data analysis software system), version 13. http://statistica.io. All tests were two-sided and tested at significant level α = 0.05.

### Outcomes

All glycemic outcomes were calculated based on the Dexcom G5 CGM readings which were stored on the cloud of Nightscout installed on Heroku cloud platform. The information regarding insulin doses was downloaded from the smartphones memories on every visit.

The primary outcome was safety of the AndroidAPS HCL system defined as: severe hypoglycemia, diabetic ketoacidosis, time spent in glycemia <54 mg/dl.

The secondary outcomes included: average sensor glycemia (SG), percentage of time in range (TIR) 70–180 mg/dl, 70–140 mg/dl, time below 70 mg/dl and above 180 mg/dl; measures of glycemic variability (Standard Deviation—SD and Coefficient of Variation—CV); changes in serum fructosamine concentration, HbA1c level, insulin requirements and body weight in study period vs run-in and baseline.

The co-secondary outcome was to describe differences in glycemic outcomes between intermittent visits during the study period, performed every 4 weeks: percentage of time below 54, below 70 mg/dl, time spent in range 70–180, 70–140 and >180 mg/dl. The secondary objective was also to describe glycemic outcomes of the last 4 weeks of the study period.

## Results

We assessed for eligibility 100 consecutive patients with type 1 diabetes visited diabetes outpatient clinic at the Department of Internal Medicine and Diabetology at the Poznan University of Medical Sciences during 3-months period (between October 2018 and January 2019). In total, 12 subjects (5 men, 7 women) were assigned to the study and completed all study procedures (Fig 1). The research was carried out sequentially in three groups (3, 4 and 5 patients) during the period from March 2019 to December 2019. In Table 1 we provide clinical characteristics of the study group. None of the subjects used APS before. During the study no episodes of severe hypoglycemia or ketoacidosis were observed. No adverse events were reported.

**Table 1:** Characteristics of the study participants.

Parameters	N = 12	95% confidence intervals
M/F	5/7	
Age, years	31.3 ± 6.7	95%CI(27.5–35.1)
Diabetes duration, years	16.1 ± 5.7	95%CI(12.9–19.3)
DDI, U/kg b.w.	0.62 ± 0.08	95%CI(0.57–0.67)
BMI, kg/m^2^	22.8 ± 2.4	95%CI(21.4–24.2)
HbA1c, %	6.8 ± 0.5	95%CI(6.5–7.1)
HbA1c, mmol/mol	51.3 ± 5.9	95%CI(48.0–54.6)
Previous insulin pump		
MiniMed^™^ Paradigm^*™*^ 715, n	1	
MiniMed^™^ Paradigm^*™*^ 722, n	1	
MiniMed^™^ Paradigm^*™*^ 754, n	5	
MiniMed^™^ 640G, n	5	
Previous CGM system		
MEDTRONIC CGM system compatible with insulin pump	11	
Dexcom G5	1	

Data are presented as N and mean ± SD. N, number; F, female; M, male; BMI, body mass index; DDI, daily dose of insulin; HbA1c, glycated hemoglobin; CGM, continuous glucose monitoring; CV, confidence interval.

Glycemic outcomes for the study period as compared to the baseline and run-in periods are presented in Table 2 as mean or median values from the evaluated period of time. The following parameters improved in the study period, presented as baseline, run-in and study period respectively: average sensor glycemia [153.3 ± 18.0, 146.4 ± 12.3, 141.4 ± 8.4 mg/dl, p<0.001], SD of sensor glycemia [54.8 ± 10.9, 49.3 ± 11.3, 46.9 ± 8.1 mg/dl, p<0.001], TIR 70-180mg/dl [68.0 ± 12.7, 74.5 ± 10.6, 79.3 ± 6.4%, p<0.001] and TIR 70–140 mg/dl [43.4 ± 13.8, 49.2 ± 10.7, 54.4 ± 7.5%, p<0.001] without increasing hypoglycemia time <54 mg/dl [0.25 (0–0.97), 0.25 (0.05–0.60), 0.35 (0.15–0.70), NS] and <70 mg/dl [2.50 (1.25–4.45), 1.85 (1.05–3.00), 1.75 (1.50–2.85), NS]. The post-hoc analysis has shown that average sensor glycemia significantly decreased in study period comparing to baseline time. Similarly, the SD of sensor glycemia significantly differed between baseline time and study period. TIR 70–140 and TIR 70–180 has significantly improved in both run-in time and study period comparing to baseline time (Table 2).

**Table 2:** Comparison of glycemic outcomes and insulin requirement in the study, run-in and baseline periods.

PARAMETER	BASELINE	RUN-IN	STUDY PERIOD	p
Average sensor glycemia, mg/dl	**153.3 ± 17.9**^b^	**146.4 ± 12.3**^a^^**,**^^b^	**141.1 ± 8.4**^a^	**=0.002**
	**95%CI(143.2–163.4)**	**95%CI(139.4–153.4)**	**95%CI(136.3–145.9)**	
Standard deviation, mg/dl	**54.8 ± 10.9**^b^	**49.3 ± 11.3**^a^^**,**^^b^	**46.9 ± 8.1**^a^	**=0.006**
	**95%CI(48.6–60.9)**	**95%CI(42.9–55.7)**	**95%CI(42.3–51.5)**	
Coefficient of Variation, %	35.7 ± 5.4	33.5 ± 5.9	33.1 ± 4.4	NS
	95%CI(32.6–38.8)	95%CI(30.2–36.8)	95%CI(30.6–35.6)	
Time <54 mg/dl, %	0.25 (0–0.97)	0.25 (0.05–0.60)	0.35 (0.15–0.70)	NS
	95%CI(0.0–1.46)*	95%CI(0.01–0.68)*	95%CI(0.11–0.78)*	
Time <70 mg/dl, %	2.50 (1.25–4.45)	1.85 (1.05–3.00)	1.75 (1.50–2.85)	NS
	95%CI(1.05–4.88)*	95%CI(0.93–3.16)*	95%CI(1.42–3.12)*	
Time 70–180 mg/dl, %	**68.0 ± 12.7**^**a**^	**74.5 ± 10.6**^b^	**79.3 ± 6.4**^b^	**<0.001**
	**95%CI(60.8–75.2)**	**95%CI(68.5–80.5)**	**95%CI(75.7–82.9)**	
Time 70–140 mg/dl, %	**43.4 ± 13.8**^**a**^	**49.2 ± 10.7**^b^	**54.4 ± 7.5**^b^	**<0.001**
	**95%CI(35.6–51.2)**	**95%CI(43.1–55.3)**	**95%CI(50.2–58.6)**	
Time >180 mg/dl, %	**28.6 ± 12.5**^b^	**22.9 ± 9.4**^b^	**18.5 ± 6.1**^**a**^	**<0.001**
	**95%CI(21.9–35.3)**	**95%CI(17.6–28.2)**	**95%CI(15.1–21.9)**	
DDI, U/kg b.w. ^#^	0.62 ± 0.08	0.59 ± 0.09	0.60 ± 0.09	NS
	95%CI(0.57–0.66)	95%CI(0.45–1.00)	95%CI(0.54–0.65)	
BMI, kg/m^2^ ^#^	22.8 ± 2.4	22.7 ± 2.6	22.9 ± 2.6	NS
	95%CI(21.4–24.2)	95%CI(21.2–24.2)	95%CI(21.4–24.4)	
Weight, kg ^#^	70.5 ± 11.8	70.6 ± 12.4	71.3 ± 12.3	NS
	95%CI(63.8–77.2)	95%CI(63.8–77.3)	95%CI(64.3–78.3)	
HbA1c, % ^#^	**6.8 ± 0.5**^c^	**6.6 ± 0.5**^b^	**6.3 ± 0.4**^**a**^	**<0.001**
	**95%CI(6.5–7.1)**	**95%CI(6.3–6.9)**	**95%CI(6.1–6.4)**	
HbA1c, mmol/mol ^#^	**51.3 ± 5.9**^c^	**48.2 ± 5.1**^b^	**45.3 ± 3.9**^**a**^	**<0.001**
	**95%CI(48.1–54.7)**	**95%CI(45.2–51.0)**	**95%CI(42.8–47.3)**	
Fructosamine, μmol/l ^#^	**351.0 ± 31.4**^b^	**327.3 ± 24.3**^**a**^	**314.6 ± 22.3**^**a**^	**<0.001**
	**95%CI(333.2–368.7)**	**95%CI(313.5–341.0)**	**95%CI(301.9–327.2)**	

p—repreated measures anova or Friedman test p-value.

^a,b,c^—post-hoc—multiple comparison analysis (Tukey’s or Dunn’s test) is presented as homogenous groups—groups with the same letters do not differ statistically significantly at α = 0.05.

*- Confidence interval for the median based on the quantiles of a binomial distribution with parameters B(n, q/100).

^#^—results presented at baseline, at the end of the run-in and at the end of the study period.

The baseline data were collected from 4 weeks, run-in phase duration was 3 weeks and study period duration was 12 weeks. Data are mean ± SD or Median (IQR); DDI: Daily Dose of Insulin, BMI: Body Mass Index, HbA1c: Glycated hemoglobin, NS: Not Significant; Repeated Measures Analysis of Variance.

The results of HbA1c value and serum fructosamine concentration are presented at baseline, at the end of the run-in and at the end of the study period. Both HbA1c and fructosamine concentration improved at the end of study period [6.8 ± 0.5, 6.6 ± 0.5, 6.3 ± 0.4, p<0.001 for HbA1c; 351.0 ± 31.4, 327.3 ± 24.3, 314.6 ± 22.3, p<0.001 for fructosamine for baseline, run-in and study period respectively]. The post-hoc analysis has shown a significant differences in HbA1c values between all analyzed time periods. The fructosamine concentration significantly decreased at both run-in time and study period comparing to baseline time (Table 2). The body weight and daily insulin dose did not change significantly at run-in and study period as compared to the baseline.

**Fig 3:**
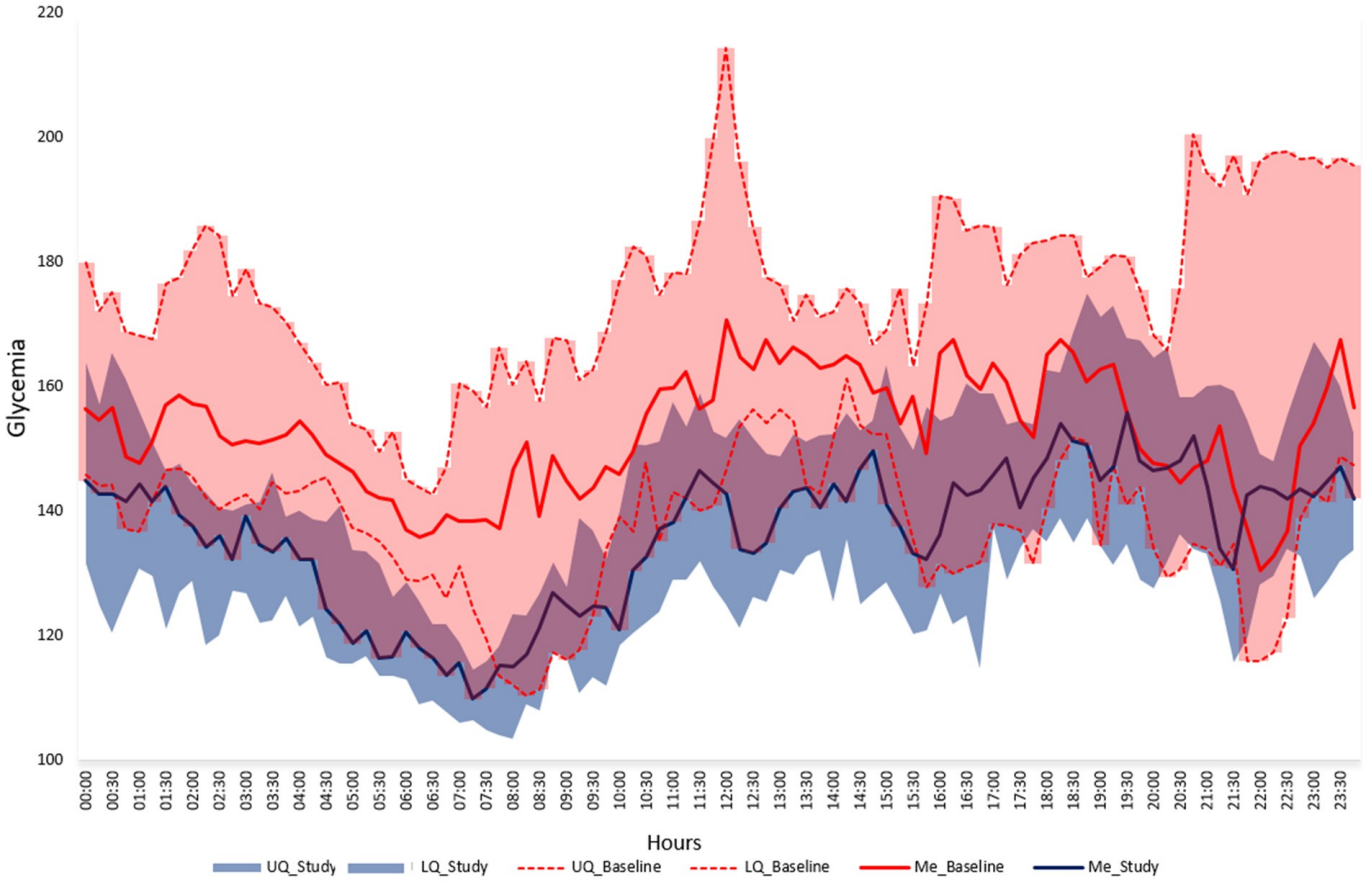
Median (IQR) glycemia in the last 4 weeks of the study period (blue area) and the baseline (pink area). UQ—upper quartile, LQ—lower quartile, Me—median.

**Fig 4:**
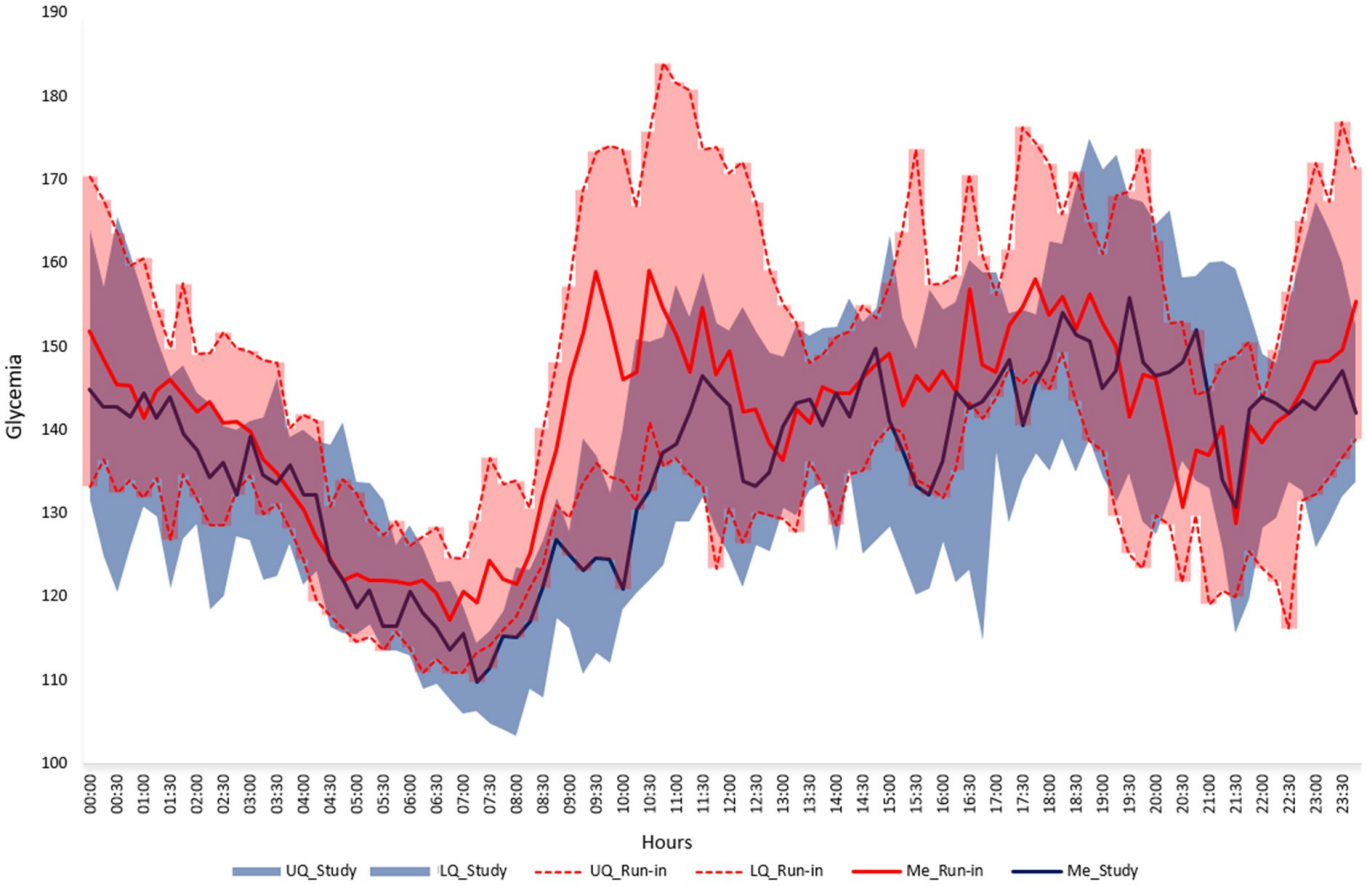
Median (IQR) glycemia in the last 4 weeks of the study period (blue area) and the run-in (pink area). UQ—upper quartile, LQ—lower quartile, Me—median.

During particular visits performed every 4 weeks in the study period average sensor glycemia, time in target range 70–180 mg/dl and 70–140 mg/dl improved significantly as well as HbA1c level (Table 3). In the last 4 weeks of the study period mean SG was 137.6 ± 8.7, percentage of time in range 70–180 mg/dl 82.1 ± 5.6%, percentage of time <54 mg/dl 0.30 (0.20–0.55) and <70 mg/dl 1.90 (1.10–3.05). The visualization of the median SG during the last 4 weeks of the study period as compared to baseline is presented in Fig 3 and as compared to run-in on Fig 4.

**Table 3:** Comparison of glycemic outcomes and insulin requirement on intermittent visits during study period.

PARAMETER	V3	V4	V5	V6	p
	EVERY 4 WEEKS				
Mean sensor glucose, mg/dl	**146.4 ± 12.3**^b^	**143.6± 11.5**^a^^**.**^^b^	**141.0 ± 7.5**^a^^**.**^^b^	**137.6 ± 8.7**^a^	**0.007**
	95%CI(139.4–153.4)	95%CI(137.1–150.1)	95%CI(136.8–145.2)	95%CI(132.7–142.5)	
Standard deviation, mg/dl	49.3 ± 11.3	47.4 ± 10.5	47.4 ± 8.0	44.1 ± 6.8	NS
	95%CI(42.9–55.7)	95%CI(41.5–53.3)	95%CI(42.9–51.9)	95%CI(40.3–47.9)	
Coefficient of Variation, %	33.5 ± 5.9	32.7 ± 5.3	33.5 ± 4.9	31.9 ± 3.7	NS
	95%CI(30.2–36.8)	95%CI(29.7–35.7)	95%CI(30.7–36.3)	95%CI(29.8–33.9)	
Time <54 mg/dl, %	0.25(0.05–0.60)	0.30(0.10–0.75)	0.35(0.15–0.55)	0.30(0.20–0.55)	NS
	95%CI(0.01–0.68)	95%CI(0.1–0.78)	95%CI(0.11–0.58)	95%CI(0.12–0.66)	
Time <70 mg/dl, %	1.85 (1.05–3.00)	1.50 (0.85–2.85)	1.65 (1.40–2.55)	1.90 (1.10–3.05)	NS
	95%CI(0.93–3.16)	95%CI(0.65–3.20)	95%CI(1.40–2.74)	95%CI(0.71–3.16)	
Time 70–180 mg/dl, %	**74.5 ± 10.6**^a^	**78.7 ± 8.1**^a^^**.**^^b^	**79.5 ± 6.3**^b^	**82.1 ± 5.6**^b^	**0.0011**
	95%CI(68.5–80.5)	95%CI(74.1–83.3)	95%CI(75.9–83.1)	95%CI(78.9–85.3)	
Time 70–140 mg/dl, %	**49.2 ± 10.7**^a^	**54.2 ± 10.8**^a^^**.**^^b^	**55.4 ± 7.1**^b^	**58.1 ± 7.6**^b^	**0.0017**
	95%CI(43.1–55.3)	95%CI(48.1–60.3)	95%CI(51.4–59.4)	95%CI(53.8–62.4)	
Time >180 mg/dl, %	**22.9 ± 9.4**^b^	**19.4 ± 7.7**^a^^**.**^^b^	**18.2 ± 5.8**^a^	**15.7 ± 5.8**^a^	**0.0014**
	95%CI(17.6–28.2)	95%CI(15.0–23.8)	95%CI(14.9–21.5)	95%CI(12.4–18.9)	
DDI, U/kg b.w.	0.73 ± 0.48	0.61 ± 0.10	0.60 ± 0.10	0.60 ± 0.10	NS
	95%CI(0.46–1.00)	95%CI(0.56–0.66)	95%CI(0.55–0.65)	95%CI(0.53–0.65)	
BMI, kg/m^2^	22.7 ± 2.6	22.9 ± 2.6	22.8 ± 2.7	22.9 ± 2.6	NS
	95%CI(21.2–24.2)	95%CI(21.4–24.4)	95%CI(21.3–24.3)	95%CI(21.4–24.4)	
Weight, kg	70.6 ± 12.4	71.1 ± 12.4	71.1 ± 12.8	71.3 ± 12.3	NS
	95%CI(63.8–77.3)	95%CI(64.1–78.1)	95%CI(63.9–78.3)	95%CI(64.3–78.3)	
HbA1c, %	**6.6 ± 0.5**^b^	**6.4 ± 0.3**^a^^**.**^^b^	**6.4 ± 0.4**^a^	**6.3 ± 0.4**^a^	**=0.005**
	95%CI(6.3–6.9)	95%CI(6.2–6.6)	95%CI(6.2–6.6)	95%CI(6.1–6.5)	
HbA1c, mmol/mol	**48.1 ± 5.2**^b^	**46.6± 3.7** ^a^^**.**^^b^	**46.0 ± 4.0**^a^	**45.3 ± 3.9**^a^	**<0.001**
	95%CI(45.2–51.0)	95%CI(44.6–48.9)	95%CI(43.3–48.3)	95%CI(42.9–47.3)	
Fructosamine, μmol/l	327.3 ± 24.3	322.0 ± 26.1	313.5 ± 20.6	314.6 ± 22.3	NS
	95%CI(313.5–341.0)	95%CI(307.2–336.8)	95%CI(301.8–325.2)	95%CI(301.9–327.2)	

p—repreated measures anova or Friedman test p-value.

^a,b,c^—post-hoc—multiple comparison analysis (Tukey’s or Dunn’s test) is presented as homogenous groups—groups with the same letters do not differ statistically significantly at α = 0.05.

*- Confidence interval for the median based on the quantiles of a binomial distribution with parameters B(n, q/100).

The visits during study period were performed every 4 weeks. Data are mean ± SD or Median (IQR); DDI: Daily Dose of Insulin, BMI: Body Mass Index, HbA1c: Glycated hemoglobin, NS: Not Significant; Repeated Measures Analysis of Variance.

The post-hoc analysis of observed power has reached value above 80% for all analyzed parameters, where the selected sample size was 12 and I-type error α = 0.05. Its values ranged from 86% for standard deviation (mg/dl) to 99% for Time 70–140 mg/dl.

## Discussion

The study showed that the use of the DIY APS based on AndroidAPS application in highly-selected, closely monitored patients with type 1 diabetes is safe. Furthermore, improvements in the metabolic control of diabetes measured with TIR parameter, average sensor glycemia and glycated hemoglobin were achieved. This is the first long-term observation over 12-weeks with the Do-It-Yourself HCL system AndroidAPS in type 1 diabetes.

The study included consecutive patients of the diabetes outpatient clinic at the Department of Internal Medicine and Diabetology at the Poznan University of Medical Sciences. The inclusion criteria were met by patients who were well educated and had experience in CSII therapy and the use of CGM. The primary endpoint was safety of the system. This issue is crucial as the system is not approved as a medical device and consists of several devices from different manufacturers, combined into a system by patients themselves. We did not report any diabetic ketoacidosis or severe hypoglycemia episodes during the study. Even though they are relatively rare nowadays, the new device is needed to be checked for the risk of acute, life-threatening complications and that also applies to very well controlled subjects. Moreover, we did not observe any technical problems with the devices used in the trial.

The secondary endpoints in our study were parameters of glycemic control, above all parameters of glycemic variability. Despite including patients with an average HbA1c of 6.8%, statistically important improvement of 0.5% was achieved. Reaching even such an improvement in HbA1c might be relevant in the context of chronic diabetes complications. Lind et al. showed in a cohort study in Sweden that the risk of retinopathy and nephropathy increased at HbA1c levels >7.0% [23]. Aiming for HbA1c <6.5% resulted in a higher risk of severe hypoglycemia. HbA1c reduction is therefore beneficial if hypoglycemia risk is not increased. In our study the median final result of HbA1c was 6.3%, achieved without an increased rate of hypoglycemia. The parameter that verifies the occurrence of hypoglycemia is percentage of time in hypoglycemic range as defined in 2017 by Danne et al. [24]. In the study, the time <54 and <70 mg/dl did not differ statistically as compared to baseline. During the study, no severe hypoglycemia was found, but it should be emphasized that no severe hypoglycemia was also found in the study group prior to the study. Use of HCL therapy leads to modest reduction of HbA1c level in well controlled type 1 diabetes. The meta-analysis of Bekiari et al. reveals the effect of 0.26% of reduction [25]. The result of 0.36% improvement was described by Tauschmann et al. [12]. Garg et al. showed a decrease in HbA1c value from 7.3% to 6.5% in adult type 1 patients on Medtronic MiniMed^™^ 670G [10]. Interestingly, the real-world data on the same HCL system published by Faulds et al. show no improvement in HbA1c level in the subgroup of patients with baseline result <7.0% [26]. Therefore, the results of our study achieved in actually well controlled patients are unique and clinically relevant. However, we are caution in comparing our results with larger studies with different artificial pancreas systems carried out on more diverse populations and often with less supervision. Further studies are necessary to verify the safety of aiming for HbA1c < 6.5% in the treatment with HCL systems.

Moreover, we have shown improvement in time in range and standard deviation results between study period and baseline. Recently TIR and other glycemic metrics, especially when measured with continuous glucose monitoring, add value as outcome in many studies. TIR was strongly associated with the risk of microvascular complications in Diabetes Control and Complications Trial (DCCT) data set presented by Beck et al. [27]. Likewise, Lu et al. in type 2 diabetes revealed significant associations between TIR and all stages of diabetic retinopathy after controlling for age, sex, BMI, diabetes duration, blood pressure, lipid profile, and HbA1c [28]. The first available and registered HCL system is Medtronic MiniMed^™^ 670G. Messer et al. indicate an increase of 14% of the time in range (70–180 mg/dl) when using the Auto Mode function after 3 months and a reduction of HbA1c by 0.75% [14]. FDA registration processes require many years of work and testing from manufacturers. Two meta-analyzes including randomized trials have shown that closed-loop systems increase the time in range by about 10% (absolute). Bekiari et al. included 40 studies both assessing single and dual hormone artificial pancreas system concluding that proportion of time in range 70–180 mg/dl was significantly higher with artificial pancreas use, both overnight (mean difference 15.15%) and over a 24 hour period (9.62%). However, the analysis might be limited by inconsistency in outcome reporting, small sample size, and short follow-up duration of individual trials [25]. Weissman et al. reports improved time in range by 12.59% with artificial pancreas systems as compared with conventional pump therapy based on the results of 24 trials. The greatest improvement of TIR was noticed for dual-hormone systems (19.52%) [29]. We have shown improvement of TIR from 68.0% before the study up to 79.3% during the 12 weeks of the study period. Therefore, the improvement of TIR using AAPS has allowed us to achieve a result comparable with the results published so far. Moreover, the coefficient of variation remained constant throughout the study and was within the recommended range [30]. Furthermore, the standard deviation was lowered. AndroidAPS operation depends on its settings which are modifiable and must be agreed when connecting devices. One of the important parameters is maximal change in the basal rates. Petruzelkova et al. in the first original research on DIY AAPS conducted during sports camp used for safety reasons a maximum increase up to 200% in the basal rates [2]. In the present study, we used maximal change in the basal up to 500% of the original basal rate.

One of the first papers on safety of HCL systems published by Bergenstal et al. drew attention to the possible increase in daily insulin dose and patients’ body weight on “artificial pancreas” [5]. Previously, weight gain achieved with intensive insulin therapy in DCCT resulted in significantly more subjects becoming overweight or obese compared with conventional treatment [31]. In the meta-analysis of Weisman et al. there was a non-significant increase in insulin dose with artificial pancreas systems compared with conventional pump therapy, especially in subgroup analyses in studies done overnight, in pediatric age groups, and with dual hormone artificial pancreas systems [29]. In our study, we did not observe increase in insulin dose or body weight with DIY AndroidAPS system. Similar results were published by Tauschmann et al. in a multicenter, 12-week randomized trial [12]. A milder system response using basal rate infusion modulation in response to hyperglycemia caused mostly by a long-absorbing meal reduces glycemic fluctuations. This can ultimately contribute to lower weight gain. Moreover, in the period between meals and/or when the meal insulin dose is too high, the algorithm suspends the basal infusion early. This ensures that no insulin is given when it is not needed. Also during physical activity insulin doses will be relatively smaller so that less additional carbohydrates and snacks are needed. However, the education of patients using DIY AP systems is crucial as these systems give greater freedom of nutrition. For some more complex meals, the patient does not need to administer a bolus insulin, and the system controls glycemia by increasing the percentage of basal rate. Protein-fat meals may not generate such hyperglycemia as on standard pump due to the system algorithm working. However, this may lead to a higher daily dose of insulin than usual, and consequently to weight gain. Finally, higher daily insulin dose on AP systems might be just related to underdosing of insulin before entering the studies due to fear of hypoglycemia. Interestingly, real-world data on Medtronic MiniMed^™^ 670G showed that the overall benefit of HCL system may vary based on baseline characteristics such as HbA1c. Faulds ER. et al. described that patients with baseline HbA1c ≥7.0% experienced an increase in weight 3 to 4 months after initiation of HCL therapy, whereas patients with baseline HbA1c <7.0% experienced weight loss [26].

The settings of AndroidAPS application were equal for the whole group and have not been modified through the observation. Of course, on the control visits the primary settings of the personal insulin pump were modified, i.e. basal rate, insulin/carbohydrate factor, insulin sensitivity factor. The results of the study show that DIY APS systems require learning and involvement from both physicians and patients’ side. The team’s greater courage to titrate the insulin dose may have contributed to the improvement in glycemic control. Moreover, patients had greater confidence in the system and less interfered with additional insulin boluses. In the last 4 weeks of the study, patients achieved the best results. We conclude that DIY AP systems require at the beginning a lot of commitment from patients who would like to start such treatment. However, over time, patients become accustomed to the devices, the algorithm and the effects they can achieve. It requires patience. Our results indicate that the learning process pays off with good results after some time. However, the learning curve for using the system was not quantified and our conclusions are based indirectly on the existing literature and achieved results [32]. It bears discussing that nowadays patients utilizing DIY systems are often doing so without the support of providers, so our data do not necessarily represent the real-world experience.

### Study limitations

The study has an observational design and was designed to test safety as the primary endpoint. As the DIY APS are not approved for use, we started with this small single arm safety study. Randomized studies are needed to compare the safety and effectiveness of different HCL systems (DIY and commercial systems). Secondly, we are aware of the fact that the included group was small number, highly selected, well-educated and motivated. This is due to the fact that CGM systems in Poland were not reimbursed for adults at the time of recruitment and were used by highly dedicated patients. We did not want to include patients without experience in the use of CGM. Finally, despite undisputable improvements during the study, we can not estimate the impact and contribution of other factors to this success. Better proficiency with sensors and pumps, close supervision by the research team and frequent medical interventions as well as patients’ engagement in the project could have influence the results. This is suggested among other by the relevant improvement in the run-in. With every continuous glucose monitoring system, the patient acquires practical experience and this is an essential and indispensable factor in the application of any closed loop system. Improved proficiency is achieved by experience and standard education [33]. Due to all these confounding factors and the observational study nature, the final improvement can not be rigorously assigned to AndroidAPS DIY system.

### Conclusions

The study revealed safety of the Do-It-Yourself HCL system AndroidAPS in adults with T1D, limited to well-controlled, highly selected and closely monitored patients. The use of AndroidAPS significantly improved HbA1c, time in range and average sensor glycemia without increasing hypoglycemia. As both patients and their medical team are gaining experience using the system over time, they improve glycemic control.

## Supporting information

S1 File Study protocol. (PDF)

S2 File Resolution of the Bioethical Committee of the Poznan University of Medical Sciences. (PDF)

S3 File Dataset. (XLS)

S4 File Log files of the statistical analyses. (PDF)
