# Supplementary material for: Safety and glycemic outcomes of do-it-yourself AndroidAPS hybrid closed-loop system in adults with type 1 diabetes
Source: PLoS One. 2021 Apr 5;16(4):e0248965. doi: 10.1371/journal.pone.0248965 (PMC8021167; doi:10.1371/journal.pone.0248965)
Supplement: S1 File — (PDF) [file pone.0248965.s001.pdf]

Assessment of safety and glycaemia during application of the hybrid closed-loop system on the basis of the AndroidAPS application.

Principal Investigators: Andrzej Gawrecki, Aleksandra Araszkiewicz

Poznan University of Medical Sciences

STUDY PROTOCOL

BACKGROUND

In the last decade, there has been an increase in the number of research projects worldwide aimed at replacing traditional personal insulin pumps with a closed-loop system called "artificial pancreas" (AP).

The Juvenile Diabetes Research Foundation (JDRF) distinguishes six levels of AP systems depending on the degree of automation, from the introduction of LGS (low-glucose suspend), through the hybrid loop (HCL - hybrid closed-loop system), requiring the patient to administer only food bolus and finally a fully closed system.

The hybrid closed-loop system consists of a CGM-continuous glucose monitoring system connected to a personal pump and integrated with an electronic receiver (application) that processes and analyzes continuous glucose data and automatically makes decisions on insulin delivery. Only the full integration of these three elements creates a closed-loop system that does not require any intervention from the user - the so-called artificial pancreas. Previous studies suggested that AP systems at level 1-3 allow for increasing the patient's residence time in the target glycemic range, reducing the frequency of hypoglycemia and improving the control of glycemia at night in comparison with the continuous subcutaneous insulin infusion (CSII) alone. The key and innovative feature in closed-loop systems is the addition of a digital control function. This allows the patient for an easier and more effective way to manage type 1 diabetes (DM1). The first commercial device constituting a hybrid closed-loop system is the MiniMed 670G system, which was approved for use by the FDA in the United States in September 2016. The system allows for automatic adjustment of the basal rate in response to glycemic fluctuations, but still requires the user to enter the amount of carbohydrates consumed and to administer boluses to meals on this basis.

In a paper recently published in *Diabetes Care*, Messer et al. describe the results of a 3-month follow-up of 31 adolescents and young adults aged 14-26 from three clinical centers using the Minimed 670G system. The authors indicate an increase of 14% of the time in range (70-180 mg/dl) when using the Auto Mode function after 3 months and a reduction of HbA1c by 0.75%. In the linear regression model, the authors showed the existence of a significant relationship between the time in

the target range and the use of the Auto Mode function. Staying 70% in the target range was associated with 75% switching on and using the Auto Mode function. (Messer et al., 2018)

Currently in the research phase, HCL systems are based on various mathematical algorithms and various types of CSII and CGM systems. In addition, they may consist of single-hormone delivery or include simultaneous administration of glucagon (dual-hormone delivery). AP systems can also be divided depending on the mathematical algorithm used: MPC (model of predictive control), PID (proportional integral derivative) or FL (fuzzy logic). The HCL system with the MPC algorithm is an OmniPod pump patch that communicates with the Dexcom G4 glucose monitoring system. The results of a brief study on the safety and efficacy of this system in children and adults with DM1 have just been published by B. Buckingham et al. in *Diabetes Technology and Therapeutics*. The study included a weekly treatment period for CSII and CGM and a 36-hour study period for the HCL system. (Buckingham et al., 2018)

Patients enrolled in our study will receive a closed-loop system consisting of the ***Dana Diabecare RS insulin pump, the Dexcom G5 continuous blood glucose monitoring system and the AndroidAPS application***. All participants will be trained in the use of these devices. The initial phase of the study using the AndroidAPS application will be carried out during hospitalization at the Department of Internal Medicine and Diabetology, Poznan University of Medical Sciences.

The AndroidAPS application was built in the Czech Republic by IT specialist Milos Kozak, representing the NightScout community. Polish representatives of NightScout also participate in the current project. The NightScout community works around the world and supports patients and diabetologists in the field of glycemic transmission via the Internet. Systems developed by them have been used in publications on, for example, telemedicine in diabetology. Remote transfer of information about treatment is one of the components of the AndroidAPS application and increases the safety of patients treated with insulin. Recently, a study on pediatric population has been carried out in the Department of Pediatrics, Second Faculty of Medicine, Charles University in Prague, by Dr. Lenka Petruzelkova (publication of Day-and-night use of Smartguard® Technology versus open-source hybrid closed-loop OpenAPS in extreme sports conditions: SLOPE STUDY in a review in *Diabetes Care*) with the active participation of the author of the application.

Dr. Bernhard Gehr from the Zentrum für Diabetes- und Stoffwechselerkrankungen Bad Heilbrunn has also extensive own experience in the use of the AndroidAPS application in daily life.

The clinical and scientific experience of these centers is available to us and constitutes the basis for further scientific research in adults. Applying the system in Europe (Czech Republic, Slovakia,

Scandinavia, Germany) is becoming more and more common. Currently, this application is independently used by about 600 people.

Operation of the algorithm in the AndroidAPS application

The basis of the AndroidAPS application is a special algorithm that, after providing all input values, is able to estimate the future change in the basal insulin needed to reach the set level. It requires the following input for proper operation:

- information on active insulin – IOB
- information on the current temporary dose – temp. basal
- current BG readings, average of last 30 minutes, average of last 10 minutes, changes since the last measurement and the trend, these data are from CGM
- patient profile information: target glycemic values, insulin sensitivity - ISF, carbohydrate absorption profile calculated by the application based on automatic modification of the basal infusion after a meal
- information about the meal, the amount of carbohydrates and insulin administrated, insulin/carbohydrate counter

Based on this information, the algorithm will predict what will happen to the patient's blood glucose within the next 30 minutes. If the result of these calculations is different from the target range of glycemia, deviation will be created. This value will be used to provide an additional correction dose of insulin.

There are no publications on the use of the AndroidAPS application in hybrid closed-loop systems in type 1 diabetes. **The planned study would be the first observation in adult patients using the HCL system based on the AndroidAPS application in outpatient settings**. It would allow for a scientific check of the operation ("feasibility") and system safety in the "real-world" setting and the impact of the system on glycemic control and satisfaction with treatment. The results of the study could be an important contribution of Poland to the progress of work on the creation of an artificial pancreas.

AIM OF RESEARCH

The aim of the study is to assess safety and glycemia during application of a hybrid closed-loop system based on the AndroidAPS application in type 1 diabetes.

Specific objectives:

Primary Objective - Evaluation of the **safety** of the closed-loop system based on AndroidAPS application, which consists in determining:

- number of hypoglycemic episodes <3.0mmol/l,
- time spent in hypoglycemia <3.0mmol/l;
- severe hypoglycemia,
- diabetic ketoacidosis

Secondary Objectives:

1. Evaluation of **metabolic control** of diabetes as a result of the use of a hybrid closed-loop system in type 1 diabetes:
    - time spent in hypoglycemia < 3.9mmol/l
    - time in range (TIR): 3.9-10 mmol/l
    - glycemic variability
    - HbA1c,
    - serum fructosamine concentration
2. Evaluation of the **effectiveness** of the basal infusion modulation in the hyperglycemia correction by the AndroidAPS system (time to achieve target glycemia 6.6mmol/l)
3. Assessment of the patients' **satisfaction** with the use of the AndroidAPS system and quality of life.

PATIENTS AND METHODS

Single-center observational study. The study will cover a group of 10 patients with type 1 diabetes under the care of the Department of Internal Medicine and Diabetology, Poznan University of Medical Sciences. The study will be carried out sequentially in two groups of 5 people.

Inclusion criteria:

1. age 18-40
2. type 1 diabetes
3. duration of diabetes > 5 years
4. treatment with a personal insulin pump from> 1 year
5. using the bolus calculator
6. experience in CGM system: using of≥70% of the time in the last 3 months
7. HbA1c <9%
8. BMI 18-30 kg / m2
9. consent to participate in the study

Exclusion criteria:

1. persistence of partial remission of type 1 diabetes
2. identified advanced chronic micro- and macroangiopathic complications of diabetes (proliferative retinopathy, diabetic kidney disease with eGFR <60 mL/min/1.73 m^2, cardiac autonomic neuropathy, coronary heart disease, stroke)
3. severe hypoglycemia in the last year
4. hypoglycemia unawareness
5. ketoacidosis in the last 3 months
6. accompanying diseases except hypothyroidism in the state of euthyreosis
7. eating disorders, depression

Visits

V0: Run-In visit – Day 0 – patient's inclusion

1. Signing of informed consent for participation in the study
2. Data collection regarding: duration of diabetes, smoking, presence of chronic complications of diabetes.
3. Complete the standardized Diabetes Satisfaction Questionnaire (DTSQs).
4. Complete the standardized Audit of Diabetes Dependent Quality of Life questionnaire **(**ADDQoL)

5. Evaluation of anthropometric data (body weight, height, waist circumference, hip circumference), body composition evaluation with TANITA, blood pressure measurement.
6. Collection of blood (10 ml) and a morning urine sample to determine: metabolic control of diabetes (HbA1c, fructosamine, lipid profile - total cholesterol, LDL cholesterol, HDL cholesterol, triglycerides), serum TSH, kidney function (creatinine level, calculation of glomerular filtration rate [eGFR] according to CKD-EPI formula, albuminuria, albumin/creatinine ratio), serum C-reactive protein concentration with highly sensitive method (hsCRP)
7. Collection of data from the last 30 days from patients' personal insulin pump and glucose meters (mean glycaemia, standard deviation, number of hypoglycemia ≤3.9mmol/l and <3.0mmol/l, daily insulin dose, basal rate)
8. Connecting the Dexcom G5 system, educating the patient about its use
9. Glucometer Contour Plus Link

V1: 1 week ± 2 days

1. data collection from pumps, meters and CGM system
2. patients' preparation procedure for connecting HCL system, first steps:
    - System visualization, analysis of the basal rate and insulin/carbohydrate ratio
    - Open system: connection to Dana Diabecare RS pumps
    - Temporal basal rate recommendations
    - Low glucose suspend function

V2: 2 weeks ± 2 days

Hospitalization at the Department of Internal Medicine and Diabetology, Poznan University of Medical Sciences, lasting **2 days± 1 days**

Analysis of patient's skills in given tasks and permission for subsequent stages of application:

- "Tuning of the closed loop", automatic change of the basal rate, gradual reduction of glycemic targets
- Subsequent correction of insulin/carbohydrate ratio and basal rate
- hyperglycemia correction function using the application - correction of the basal infusion instead of correction bolus

- Administration of boluses only by introducing carbohydrates consumed

The patient receives the AndroidAPS application, which is fully activated in the hospital setting and starts functioning in home conditions for **7 ± 2 days**. All this time, patients have the option of telephone contact with a research team.

V3: 3 weeks ± 2 days

Technical control of HCL system and check-up of patient's skills.

1. Evaluation of anthropometric data (body weight, height, waist circumference, hip circumference), body composition evaluation with TANITA, blood pressure measurement.

2. Collection of blood (10 ml): metabolic control of diabetes (HbA1c, fructosamine, lipid profile - total cholesterol, LDL cholesterol, HDL cholesterol, triglycerides), serum C-reactive protein concentration with highly sensitive method (hsCRP)

START OF THE STUDY OBSERVATION FOR 12 WEEKS.

V4: 7 weeks ± 2 days

1. data collection from pumps, meters, CGM systems

3. Collection of blood (10 ml): metabolic control of diabetes (HbA1c, fructosamine, lipid profile - total cholesterol, LDL cholesterol, HDL cholesterol, triglycerides), serum C-reactive protein concentration with highly sensitive method (hsCRP)

2. Evaluation of anthropometric data (body weight, height, waist circumference, hip circumference), body composition evaluation with TANITA, blood pressure measurement.

3. Complete the standardized Diabetes Satisfaction Questionnaire (DTSQs).

V5: 11 weeks ± 2 days

4. data collection from pumps, meters, CGM systems

4. Collection of blood (10 ml): metabolic control of diabetes (HbA1c, fructosamine, lipid profile - total cholesterol, LDL cholesterol, HDL cholesterol, triglycerides), serum C-reactive protein concentration with highly sensitive method (hsCRP)

5. Evaluation of anthropometric data (body weight, height, waist circumference, hip circumference), body composition evaluation with TANITA, blood pressure measurement.

6. Complete the standardized Diabetes Satisfaction Questionnaire (DTSQs).

V6: 15 weeks ± 2 days

1. data collection from pumps, meters, CGM systems
2. Collection of blood (10 ml) and a morning urine sample to determine: metabolic control of diabetes (HbA1c, fructosamine, lipid profile - total cholesterol, LDL cholesterol, HDL cholesterol, triglycerides), serum TSH, kidney function (creatinine level, calculation of glomerular filtration rate [eGFR] according to CKD-EPI formula, albuminuria, albumin/creatinine ratio), serum C-reactive protein concentration with highly sensitive method (hsCRP)
3. Evaluation of anthropometric data (body weight, height, waist circumference, hip circumference), body composition evaluation with TANITA, blood pressure measurement.
4. Complete the standardized Diabetes Satisfaction Questionnaire (DTSQs and DTSQc).
5. Complete the standardized Audit of Diabetes Dependent Quality of Life questionnaire **(**ADDQoL)

PATIENT SAFETY DURING THE RESEARCH:

- possibility of telephone contact with researchers 24 hours a day
- possibility of hospital admission to the department of internal medicine and diabetology
- the use of continuous glucose monitoring system
- obligatory self-control on glucometer min. 4-6 measurements per day
- the ability to turn off the application at any time and to administer insulin via an insulin pump in standard mode
- if the CGM-application-insulin pump is not communicated, the insulin pump automatically switches to the standard mode

Statistical analysis will be carried out using the Statistica program. The tests of descriptive and comparative statistics will be used. In evaluating the relationship between variables, correlation and multivariate regression tests will be used. The results will be compared between the V3 visits and V4-V6.

EXPECTED RESULTS

1. Demonstration of the safety of the hybrid closed-loop system in adults with type 1 diabetes.

2. Demonstration of the improvement in metabolic control of diabetes mellitus with HCL system based on the AndroidAPS application in type 1 diabetes (improvement in HbA1c, fructosamine concentrations, reduction of time in hypoglycemia, increase / prolongation of the time in range).
3. Demonstration of the effectiveness of the basal infusion modulation in the hyperglycemia correction by the AndroidAPS system.
4. Demonstration of the improvement in patients' satisfaction with HCL system based on the AndroidAPS application as well as quality of life.

Duration: 15 weeks for every patient (3 weeks RUN-IN and 12 weeks observation)

THE IMPLICATIONS OF THE PROJECT AND ITS RESULTS FOR THE DIAGNOSIS AND TREATMENT OF DIABETES AND ITS COMPLICATIONS:

The implementation of traditional insulin pumps and continuous monitoring systems in the treatment of type 1 diabetes did not allow to achieve satisfactory metabolic control in different age groups. In addition, severe, and above all, mild hypoglycemia episodes have not been eliminated. Treatment of diabetes still constitutes a high psychological burden for patients, resulting in poorer quality of life, but also three times more frequent depression and suicide episodes than in the healthy population. The obtained results of treatment are also caused by a deficit in diabetes education, lack of funding of education in Poland and many other countries. Patterns in insulin dosage are reproducible in each patient, and the number of potential solutions in daily life is limited, therefore, the goal is to create a computer algorithm that will independently decide on the dosage of insulin.

The AndroidAPS application, which is made available to more and more patients, for example in the Czech Republic and Germany, opens up new possibilities in the treatment of type 1 diabetes. The creation and implementation of the project is dealt with by IT specialists, technologists who represent the worldwide community of parents of children with diabetes called NightScout. The IT products they built to enable remote monitoring of glycaemia via the Internet cloud are also commonly used in Poland. However, they are implemented without the participation of diabetologists and scientists, the users are fully responsible for their functioning. So far, Nightscout's initiatives and projects have operated in parallel to the pharmaceutical industry, although Joyce M.

Lee et al. in JAMA (2016) emphasizes that their innovative products open a new era in medicine. (Lee et al., 2016).

Therefore, it is required to determine the safety and effectiveness of the algorithm that controls insulin delivery through an insulin pump synchronized with CGM. The presented study will allow diabetologists to evaluate the safety and effectiveness of the algorithm, which will be rapidly disseminated by patients. It is particularly important to determine the patients' interventions, their number and situations in which they are necessary during the automatic operation of the system. In addition, the statistical calculation of glycemic results together with the assessment of its variability will help to improve the AndroidAPS algorithm. This development will consist of clinical observations and consideration of additional factors influencing glycaemia. The development of logistics and phases of safe implementation of the artificial pancreas and the supervision of the patient is also important.

Publication of the obtained results would be the first Polish report regarding the artificial pancreas. Its introduction to clinical practice will change the lives of patients with type 1 diabetes and give better treatment outcomes and reduce chronic complications of the disease.

REFERENCES:

1. Messer LH, Forlenza GP, Sherr JL, Wadwa RP, Buckingham BA, Weinzimer SA, Maahs DM, Slover RH. Optimizing Hybrid Closed-Loop Therapy in Adolescents and Emerging Adults Using the MiniMed 760G System. Diabetes Care 2018 Feb 14. Ahead of print.

2. Iturralde E, Tanenbaum ML, Hanes SJ, Suttiratana SC, Ambrosino JM, Ly TT, Maahs DM, Naranjo D, Walders-Abramson N, Weinzimer SA, Buckingham BA, Hood KK. Expectations and Attitudes of Individuals With Type 1 Diabetes After Using a Hybrid Closed Loop System. Diabetes Educ. 2017; 43(2): 223-232.

3. Buckingham BA, Forlenza GP, Pinsker JE, Christiansen MP, Wadwa RP, Schneider J, Peyser TA, Dassau E, Lee JB, O'Connor J, Layne JE, Ly TT. Safety and Feasibility of the OmniPod Hybrid Closed-Loop System in Adult, Adolescent, and Pediatric Patients with Type 1 Diabetes Using a Personalized Model Predictive Control Algorithm. Diabetes Technol Ther. 2018 Feb 12. doi: 10.1089/dia.2017.0346. [Epub ahead of print].

4. Trevitt S, Simpson S, Wood A. Artificial Pancreas Device Systems for the Closed-Loop Control of Type 1 Diabetes: What Systems Are in Development? J Diabetes Sci Technol. 2016; 10(3): 714-23

5. Bergenstal RM, Garg S, Weinzimer SA, et al.: Safety of a hybrid closed-loop insulin delivery system in patients with type 1 diabetes. JAMA 2016;316:1407–1408.

6. Garg SK, Weinzimer SA, Tamborlane WV, et al.: Glucose outcomes with the in-home use of a hybrid closed-loop insulin delivery system in adolescents and adults with type 1 diabetes. Diabetes Technol Ther 2017;19:155–163.

7. Weisman A, Bai JW, Cardinez M, et al.: Effect of artificial pancreas systems on glycaemic control in patients with type 1 diabetes: a systematic review and meta-analysis of outpatient randomised controlled trials. Lancet Diabetes Endocrinol 2017;5:501–512

8. Lee JM\, Hirschfeld E, Wedding J. A Patient-Designed Do-It-Yourself Mobile Technology System for Diabetes: Promise and Challenges for a New Era in Medicine. JAMA. 2016 Apr 12;315(14):1447-8. doi: 10.1001/jama.2016.1903.

9. El-Khatib FH, Balliro C, Hillard MA, Magyar KL, Ekhlaspour L, Sinha M, Mondesir D, Esmaeili A, Hartigan C, Thompson MJ, Malkani S, Lock JP, Harlan DM, Clinton P, Frank E, Wilson DM, DeSalvo D, Norlander L, Ly T, Buckingham BA, Diner J, Dezube M, Young LA, Goley A, Kirkman MS, Buse JB, Zheng H, Selagamsetty RR, Damiano ER, Russell SJ. Home use of a bihormonal bionic pancreas versus insulin pump therapy in adults with type 1 diabetes: a multicentre randomised crossover trial. Lancet. 2017 Jan 28;389(10067):369-380.
