# Supplementary material for: Safety and glycemic outcomes of do-it-yourself AndroidAPS hybrid closed-loop system in adults with type 1 diabetes
Source: PLoS One. 2021 Apr 5;16(4):e0248965. doi: 10.1371/journal.pone.0248965 (PMC8021167; doi:10.1371/journal.pone.0248965)
Supplement: S2 File — (PDF) [file pone.0248965.s002.pdf]

Katarzyna Kordas
Court interpreter and certified translator of the English language
based in Poznań, Poland

CERTIFIED TRANSLATION FROM THE POLISH LANGUAGE

[The document to be translated consists of 2 pages stapled together. Translator's notes are provided in italics and square brackets.]

POZNAN UNIVERSITY OF MEDICAL SCIENCES

BIOETHICAL COMMITTEE OF THE POZNAN UNIVERSITY OF MEDICAL SCIENCES

Collegium Stomatologicum
ul. Bukowska 70
60-812 Poznań

tel. (+48 61) 854 73 36
www.bioetyka.ump.edu.pl

Resolution no. 710/18

Pursuant to the Act on the Professions of Physicians and Dentists dated 5 December 1996 (consolidated text: Journal of Laws of 2017, item 125, as amended), Regulation of the Minister of Health and Social Care dated 11 May 1999 concerning detailed rules of the appointment, financing and manner of operation of bioethical committees (Journal of Laws no. 47, item 480); Pharmaceutical Law Act dated 6 September 2001 (consolidated text: Journal of Laws of 2016, item 2142, as amended); Regulation of the Minister of Finance dated 30 April 2004 concerning mandatory third party liability insurance of the researcher and the sponsor (Journal of Laws of 2004, no. 101, item 1034, as amended); Regulation of the Minister of Finance dated 18 May 2005 amending the Regulation concerning mandatory third party liability insurance of the researcher and the sponsor (Journal of Laws no. 101, item 845); Regulation of the Minister of Health dated 30 April 2004 concerning the manner of conducting clinical research with the participation of minors (Journal of Laws of 2004, no. 104, item 1108); Regulation of the Minister of Health dated 30 April 2004 concerning reporting of a unexpected serious adverse reaction to the medicinal product (Journal of Laws no. 104, item 1107); Regulation of the Minister of Health dated 17 February 2016 concerning model applications related to the clinical research of a medical device or an active medical device to be implanted and the amount of fees for filing such applications (Journal of Laws of 2016, item 208); Medical Device Act dated 20 May 2010 (consolidated text: Journal of Laws of 2017, item 211, as amended); Regulation of the Minister of Finance dated 6 October 2010 concerning mandatory third party liability insurance of the sponsor and the clinical researcher in relation to the clinical research of devices (Journal of Laws of 2010, no. 194, item 1290); Act on the Office for Registration of Medicinal Products, Medical Devices and Biocidal Products dated 18 March 2011 (consolidated text: Journal of Laws of 2016, item 1718); Regulation of the Minister of Health dated 2 May 2012 concerning the Good Clinical Practice (Journal of Laws of 2012, item 489); Regulation of the Minister of Health dated 2 May 2012 concerning model applications submitted in relation to the clinical research of a medicinal product as well as the amount and method of payment of fees for filing an application for the clinical research commencement (Journal of Laws of 2012, no. 0, item 491); based on the Declaration of Helsinki – Ethical Principles for Medical Research Involving Human Subjects and provisions of ICH GCP.

At the meeting held on 14 June 2018, the Bioethical Committee considered a scientific research application.

Project managers: ***Andrzej Gawrecki, MD, PhD***
Aleksandra Araszkiewicz, MD, PhD, Assoc. Professor

Research place: ***Department of Internal Medicine and Diabetology***
Poznan University of Medical Sciences

Principal researcher: ***Andrzej Gawrecki, MD, PhD***

Research team members: ***Aleksandra Araszkiewicz, MD, PhD, Assoc. Professor; Anna Adamska, MD; Urszula Frąckiowiak, MD; Katarzyna Ksiądz, MD; Monika Pietrzak, MA; Dominik Powęzki, BA***

Supervisor: Professor Dorota Zozulińska-Ziółkiewicz, MD, PhD

Research subject:
"Assessment of safety and glycaemia during application of the hybrid closed-loop system on the basis of the AndroidAPS application".

The Committee adopted the resolution approving the application.

[Name stamp]
Chairman of the Committee
[illegible signature]
Professor Paweł Chęciński, MD, PhD

KATARZYNA KORDAS TŁUMACZ PRZYSIĘGŁY JĘZYKA ANGIELSKIEGO NR TP/2352/[illegible]

Signatures of the members of the Bioethical Committee adopting Resolution no. 710/18 dated 14 June 2018.

No.	Name and surname	Specialty	Place of work	Signature
1.	Chairman of the Committee<br>**Professor Paweł Chęciński**	general and vascular surgery and angiology	Department of General and Vascular Surgery and Angiology of the Poznan University of Medical Sciences, Health Care Centre of the Ministry of Internal Affairs and Administration, ul. Dojazd 34, 60-631 Poznań	*[illegible signature]*
2.	Vice Chairman of the Committee<br>**Professor Janusz Wiśniewski**	philosopher	Faculty of Political Science and Journalism of the Adam Mickiewicz University in Poznań, ul. Umultowska 89A, 61-614 Poznań	*[illegible signature]*
3.	Professor Zygmunt Adamski	dermatology and venereology	Chair and Department of Dermatology of the Poznan University of Medical Sciences, ul. Przybyszewskiego 49, 60-355 Poznań	*[illegible signature]*
4.	Krystyna Babiak, LLD	lawyer	Office of the Attorney at Law, Krystyna Babiak LLD, ul. Czartoria 1/2, 61-102 Poznań	*[illegible signature]*
5.	Priest Andrzej Bohdanowicz, Professor of the Adam Mickiewicz University	theology	Faculty of Theology of the Adam Mickiewicz University in Poznań, ul. Wieżowa 2/4, Poznań	*[illegible signature]*
6.	Professor Maciej Krawczyński	clinical genetics, ophthalmology	Chair and Unit of Medical Genetics of the Poznan University of Medical Sciences, ul. Rokietnicka 8, 60-806 Poznań	*[illegible signature]*
7.	Jolanta Łojko-Kołodziejczak, MA	nurse	Ward Nurse of the Paediatrics Reception Room of Karol Jonscher Hospital of the Poznan University of Medical Sciences, ul. Szpitalna 27/33, 60-572 Poznań	*[illegible signature]*
8.	Krystyna Malinger, MA	pharmacist	Pharmacy at the Gynaecology and Obstetrics Hospital of the Poznan University of Medical Sciences, ul. Polna 33, 60-535 Poznań	*[illegible signature]*
9.	Professor Andrzej Marszałek	anatomical pathology	Chair and Unit of Cancer Pathology and Prevention of the Poznan University of Medical Sciences, ul. Garbary 15, 61-866 Poznań	*[illegible signature]*
10.	Professor Maciej Owecki	internal medicine, endocrinology	Public Health Division of the Poznan University of Medical Sciences, ul. Dąbrowskiego 79, 60-529 Poznań	*[illegible signature]*
11.	Professor Wojciech Służewski	paediatrics, child neurology, infectious diseases	Department of Infectious Diseases and Child Neurology of the Poznan University of Medical Sciences, ul. Szpitalna 27/33, 60-572 Poznań	*[illegible signature]*
12.	Professor Robert Spaczyński	gynaecology and obstetrics	Department of Infertility and Reproductive Endocrinology of the Poznan University of Medical Sciences, ul. Polna 33, 60-535 Poznań	*[illegible signature]*
13.	Piotr Tomczak, MD, PhD	clinical oncology, radiotherapy	Department of Oncology of the Poznan University of Medical Sciences, ul. Szamarzewskiego 82/84, 60-569 Poznań	*[illegible signature]*
14.	Professor Joanna Twarowska-Hauser	psychiatry	Department of Adult Psychiatry, Genetics in Psychiatry Unit of the Poznan University of Medical Sciences, ul. Rokietnicka 8, 60-806 Poznań	*[illegible signature]*
15.	Professor Henryk Wysocki	internal medicine, cardiology	Mieszko I School of Pedagogy and Administration in Poznań, ul. Bułgarska 55, 60-320 Poznań	*[illegible signature]*

KATARZYNA KORDAS TŁUMACZ PRZYSIĘGŁY JĘZYKA ANGIELSKIEGO NR TP/2352/05

Rep. no. 63/2018
I hereby certify that this is a true and fair translation of the original document in the Polish language submitted to me.

Katarzyna Kordas

Poznań, 29 June 2018
