# Supplementary material for: Safety and glycemic outcomes of do-it-yourself AndroidAPS hybrid closed-loop system in adults with type 1 diabetes
Source: PLoS One. 2021 Apr 5;16(4):e0248965. doi: 10.1371/journal.pone.0248965 (PMC8021167; doi:10.1371/journal.pone.0248965)
Supplement: S4 File — (PDF) [file pone.0248965.s004.pdf]

Variable	Descriptive Statistics (Dataset_29.01)						
	Valid N	Mean	Median	Minimum	Maximum	Lower Quartile	Upper Quartile
age [years]	12	31,33333	30,50000	22,00000	42,00000	26,50000	37,50000

Variable	Descriptive Statistics (Dataset_29.01)
	Std.Dev.
age [years]	6,651498

Category	Frequency table: sex (m=1) (Dataset_29.01)			
	Count	Cumulative Count	Percent	Cumulative Percent
0	7	7	58,33333	58,3333
1	5	12	41,66667	100,0000
Missing	0	12	0,00000	100,0000

Variable	Descriptive Statistics (Dataset_29.01)			
	Valid N	Mean	Median	Minimum
V0 weight [kg]	12	70,5333	66,8000	52,0000
V0 height [cm]	12	175,8333	177,0000	160,0000
V0 BMI [kg/m2]	12	22,7583	22,2500	20,1000
V0 HbA1c [%]	12	6,8417	6,8000	6,2000
V0 HbA1c [mmol/l]	12	51,2794	50,8240	44,2660
V0 fructosamine [umol/l]	11	351,0000	330,0000	324,0000
V0 mean sensor glucose 4 weeks [mg/dl]	12	153,3333	151,5000	128,0000
V0 SD sensor glucose 4 weeks[mg/dl]	12	54,8333	54,0000	36,0000
V0 CV sensor glucose 4 weeks [%]	12	35,6845	35,4687	28,1250
V0 DDI 4 weeks [U]	12	43,5592	42,3450	28,2000
V0 DDI 4 weeks [U/kg b.w.]	12	0,6166	0,5884	0,5417
V0 time <54 mg/dl 4 weeks [%]	12	0,9108	0,2500	0,0000
V0 time <70 mg/dl 4 weeks [%]	12	3,3183	2,5000	0,0000
V0 time 70-180 mg/dl 4 weeks [%]	12	68,0433	62,8350	53,5000
V0 time 70-140 mg/dl 4 weeks [%]	12	43,3925	35,0550	30,5000
V0 time >180 mg/dl 4 weeks [%]	12	28,6383	32,0800	8,2500

Variable	Descriptive Statistics (Dataset_29.01)			
	Maximum	Lower Quartile	Upper Quartile	Std.Dev.
V0 weight [kg]	99,5000	64,4000	75,5000	11,77751
V0 height [cm]	194,0000	165,5000	182,5000	11,62156
V0 BMI [kg/m2]	26,4000	20,7000	25,0500	2,40282
V0 HbA1c [%]	8,0000	6,5000	6,9500	0,53676
V0 HbA1c [mmol/l]	63,9400	47,5450	52,4635	5,86673

Variable	Descriptive Statistics (Dataset_29.01)			
	Maximum	Lower Quartile	Upper Quartile	Std.Dev.
V0 fructosamine [umol/l]	410,0000	326,0000	386,0000	31,40701
V0 mean sensor glucose 4 weeks [mg/dl]	175,0000	136,5000	171,0000	17,99663
V0 SD sensor glucose 4 weeks[mg/dl]	73,0000	47,5000	64,0000	10,92814
V0 CV sensor glucose 4 weeks [%]	44,7368	31,6272	38,4376	5,35555
V0 DDI 4 weeks [U]	60,4000	37,6800	49,3000	9,21423
V0 DDI 4 weeks [U/kg b.w.]	0,8043	0,5581	0,6570	0,07893
V0 time <54 mg/dl 4 weeks [%]	4,2500	0,0000	0,9650	1,56564
V0 time <70 mg/dl 4 weeks [%]	9,5000	1,2500	4,4500	2,95004
V0 time 70-180 mg/dl 4 weeks [%]	89,5000	57,5000	78,6250	12,66828
V0 time 70-140 mg/dl 4 weeks [%]	65,5000	32,2500	56,8000	13,78945
V0 time >180 mg/dl 4 weeks [%]	43,0000	17,2500	39,1250	12,52273

Variable	Descriptive Statistics (Dataset_29.01)					
	Valid N	Mean	Median	Minimum	Maximum	Lower Quartile
V3 weight [kg]	12	70,6167	66,7500	49,2000	101,3000	65,1000
V3 height [cm]	12	176,0833	177,0000	160,0000	194,0000	167,0000
V3 BMI [kg/m2]	12	22,7000	22,1000	19,0000	26,9000	20,7000
V3 HbA1c [%]	12	6,5583	6,5500	6,0000	7,3000	6,1500
V3 HbA1c [mmol/l]	12	48,1826	48,0915	42,0800	56,2890	43,7195
V3 fructosamine [umol/l]	12	327,3333	324,5000	295,0000	387,0000	310,0000
V3 SD DDI [U]	12	5,6867	5,7500	2,9900	8,1600	3,6300
V3 mean DDI [U]	12	42,3383	41,1100	20,1100	60,2600	38,9250
V3 DDI [U/kg b.w.]	12	0,5945	0,5960	0,4087	0,7708	0,5502
V3 mean glucose [mg/dl]	12	146,3500	146,1500	128,0000	162,8000	135,6000
V3 median glucose [mg/dl]	12	138,3333	139,5000	123,0000	154,0000	127,5000
V3 SD glucose [mg/dl]	12	49,3417	47,8500	31,5000	70,3000	41,6500
V3 CV glucose [%]	12	33,4805	32,9942	24,6094	45,5016	29,6616
V3 time 70-180 mg/dl [%]	12	74,4500	75,5500	58,4000	91,9000	65,9000
V3 time 70-140 mg/dl [%]	12	49,1750	48,3000	35,3000	66,5000	39,6000
V3 time <70 mg/dl [%]	12	2,6083	1,8500	0,2000	9,5000	1,0500
V3 time <54 mg/dl [%]	12	0,7000	0,2500	0,0000	3,8000	0,0500
V3 time >180 mg/dl [%]	12	22,9167	23,0000	6,9000	33,2000	14,5500

Variable	Descriptive Statistics (Dataset_29.01)	
	Upper Quartile	Std.Dev.
V3 weight [kg]	75,7500	12,41413
V3 height [cm]	182,5000	11,40541
V3 BMI [kg/m2]	25,1000	2,59019
V3 HbA1c [%]	6,9000	0,46799
V3 HbA1c [mmol/l]	51,9170	5,11514
V3 fructosamine [umol/l]	339,5000	24,30987
V3 SD DDI [U]	7,6950	2,02169
V3 mean DDI [U]	47,2450	10,77291
V3 DDI [U/kg b.w.]	0,6276	0,09539
V3 mean glucose [mg/dl]	156,3500	12,31241
V3 median glucose [mg/dl]	146,0000	10,80684

Variable	Descriptive Statistics (Dataset_29.01)	
	Upper Quartile	Std.Dev.
V3 SD glucose [mg/dl]	57,3000	11,32595
V3 CV glucose [%]	36,3888	5,91027
V3 time 70-180 mg/dl [%]	83,8500	10,55449
V3 time 70-140 mg/dl [%]	58,6500	10,69002
V3 time <70 mg/dl [%]	3,0000	2,58332
V3 time <54 mg/dl [%]	0,6000	1,14970
V3 time >180 mg/dl [%]	32,0000	9,37791

Variable	Descriptive Statistics (Dataset_29.01)					
	Valid N	Mean	Median	Minimum	Maximum	Lower Quartile
V4 weight [kg]	12	71,1417	67,9000	49,6000	101,1000	64,9500
V4 height [cm]	12	176,0833	177,0000	160,0000	194,0000	167,0000
V4 BMI [kg/m2]	12	22,8500	22,2000	19,1000	26,9000	20,7000
V4 HbA1c [%]	12	6,4167	6,5000	5,8000	6,9000	6,1500
V4 HbA1c [mmol/l]	12	46,6342	47,5450	39,8940	51,9170	43,7195
V4 fructosamine [umol/l]	12	322,0000	321,5000	285,0000	375,0000	299,5000
V4 SD DDI [U]	12	6,4133	6,0350	2,3700	12,7300	3,8850
V4 mean DDI [U]	12	43,6300	42,1200	19,0700	62,1500	39,8850
V4 DDI [U/kg b.w.]	12	0,6070	0,6190	0,3845	0,7993	0,5646
V4 mean glucose [mg/dl]	12	143,5833	144,6000	120,2000	158,7000	138,6500
V4 median glucose [mg/dl]	12	135,1667	136,5000	114,0000	150,0000	128,5000
V4 SD glucose [mg/dl]	12	47,3750	47,1000	30,2000	64,4000	40,5500
V4 CV glucose [%]	12	32,7311	32,8486	23,9113	41,9817	29,0895
V4 time 70-180 mg/dl [%]	12	78,7000	78,5000	65,1000	93,9000	72,1500
V4 time 70-140 mg/dl [%]	12	54,2000	51,1000	37,4000	74,5000	48,9000
V4 time <70 mg/dl [%]	12	1,9167	1,5000	0,2000	4,2000	0,8500
V4 time <54 mg/dl [%]	12	0,5083	0,3000	0,0000	1,5000	0,1000
V4 time >180 mg/dl [%]	12	19,3833	20,2000	4,8000	30,7000	15,4000

Variable	Descriptive Statistics (Dataset_29.01)	
	Upper Quartile	Std.Dev.
V4 weight [kg]	77,1000	12,43700
V4 height [cm]	182,5000	11,40541
V4 BMI [kg/m2]	25,3500	2,60995
V4 HbA1c [%]	6,6000	0,34068
V4 HbA1c [mmol/l]	48,6380	3,72360
V4 fructosamine [umol/l]	336,5000	26,06286
V4 SD DDI [U]	8,6350	3,31250
V4 mean DDI [U]	48,3700	11,42098
V4 DDI [U/kg b.w.]	0,6419	0,09997
V4 mean glucose [mg/dl]	153,5500	11,54720
V4 median glucose [mg/dl]	141,5000	10,38209
V4 SD glucose [mg/dl]	54,3500	10,52833
V4 CV glucose [%]	35,8172	5,29350
V4 time 70-180 mg/dl [%]	83,4000	8,14203

Variable	Descriptive Statistics (Dataset_29.01)	
	Upper Quartile	Std.Dev.
V4 time 70-140 mg/dl [%]	61,1000	10,83110
V4 time <70 mg/dl [%]	2,8500	1,33949
V4 time <54 mg/dl [%]	0,7500	0,54848
V4 time >180 mg/dl [%]	25,5000	7,71172

Variable	Descriptive Statistics (Dataset_29.01)					
	Valid N	Mean	Median	Minimum	Maximum	Lower Quartile
V5 weight [kg]	12	71,0917	68,5000	49,6000	101,900	64,0500
V5 height [cm]	12	176,0833	177,0000	160,0000	194,000	167,0000
V5 BMI [kg/m2]	12	22,8333	22,2500	19,1000	27,100	20,8000
V5 HbA1c [%]	12	6,3583	6,3500	5,7000	7,000	6,1500
V5 HbA1c [mmol/l]	12	45,9966	45,9055	38,8010	53,010	43,7195
V5 fructosamine [umol/l]	11	313,5455	315,0000	285,0000	347,0000	290,0000
V5 SD DDI [U]	12	6,7308	6,4750	3,2100	10,840	4,7450
V5 mean DDI mean [U]	12	43,2858	42,3550	20,2100	62,020	35,8800
V5 DDI [U/kg b.w.]	12	0,6009	0,6079	0,4075	0,768	0,5445
V5 mean glucose [mg/dl]	12	141,0417	141,6500	129,3000	151,100	135,0500
V5 median glucose [mg/dl]	12	132,6667	133,0000	121,0000	143,000	127,5000
V5 SD glucose [mg/dl]	12	47,4000	48,4000	35,1000	62,600	41,1500
V5 CV glucose [%]	12	33,5275	32,7261	26,5507	44,209	30,3837
V5 time 70-180 mg/dl [%]	12	79,5333	78,2000	68,3000	88,600	75,2500
V5 time 70-140 mg/dl [%]	12	55,3917	51,7000	48,5000	68,400	50,1500
V5 time <70 mg/dl [%]	12	2,3083	1,6500	0,3000	8,400	1,4000
V5 time <54 mg/dl [%]	12	0,5000	0,3500	0,0000	2,600	0,1500
V5 time >180 mg/dl [%]	12	18,1583	19,9500	8,6000	24,400	13,2500

Variable	Descriptive Statistics (Dataset_29.01)	
	Upper Quartile	Std.Dev.
V5 weight [kg]	77,8500	12,8118
V5 height [cm]	182,5000	11,4054
V5 BMI [kg/m2]	25,4500	2,6905
V5 HbA1c [%]	6,6000	0,3704
V5 HbA1c [mmol/l]	48,6380	4,0485
V5 fructosamine [umol/l]	322,0000	20,59303
V5 SD DDI [U]	8,4350	2,5544
V5 mean DDI mean [U]	53,4450	12,1077
V5 DDI [U/kg b.w.]	0,6761	0,0989
V5 mean glucose [mg/dl]	147,2500	7,4729
V5 median glucose [mg/dl]	140,0000	7,5959
V5 SD glucose [mg/dl]	52,4000	7,9720
V5 CV glucose [%]	36,2139	4,9335
V5 time 70-180 mg/dl [%]	85,2500	6,3464
V5 time 70-140 mg/dl [%]	58,8500	7,0693
V5 time <70 mg/dl [%]	2,5500	2,1034
V5 time <54 mg/dl [%]	0,5500	0,6980

Variable	Descriptive Statistics (Dataset_29.01)	
	Upper Quartile	Std.Dev.
V5 time >180 mg/dl [%]	23,2000	5,8082

Variable	Descriptive Statistics (Dataset_29.01)					
	Valid N	Mean	Median	Minimum	Maximum	Lower Quartile
V6 weight [kg]	12	71,3083	69,4500	49,2000	100,1000	65,0000
V6 height [cm]	12	176,0833	177,0000	160,0000	194,0000	167,0000
V6 BMI [kg/m2]	12	22,9167	22,9000	19,0000	26,6000	20,8500
V6 HbA1c [%]	12	6,2917	6,3000	5,9000	7,1000	6,0000
V6 HbA1c [mmol/l]	12	45,2679	45,3590	40,9870	54,1030	42,0800
V6 fructosamine [umol/l]	12	314,5833	315,0000	286,0000	353,0000	296,0000
V6 SD DDI [U]	12	6,8000	6,4550	2,9500	11,1800	4,4650
V6 mean DDI [U]	12	43,1525	41,5250	20,2500	62,3200	36,2500
V6 DDI [U/kg b.w.]	12	0,5985	0,5838	0,4116	0,7990	0,5288
V6 mean glucose [mg/dl]	12	137,6250	140,2500	123,1000	148,6000	128,6000
V6 median glucose [mg/dl]	12	129,9167	131,0000	119,0000	139,0000	122,0000
V6 SD glucose [mg/dl]	12	44,0833	44,3500	32,7000	59,1000	39,5000
V6 CV glucose [%]	12	31,9267	31,3327	26,5638	40,8149	29,8720
V6 time 70-180 mg/dl [%]	12	82,1000	80,9000	71,9000	91,6000	79,0500
V6 time 70-140 mg/dl [%]	12	58,0667	56,7000	46,6000	70,1000	52,5000
V6 time <70 mg/dl [%]	12	2,1917	1,9000	0,2000	5,5000	1,1000
V6 time <54 mg/dl [%]	12	0,4833	0,3000	0,0000	2,0000	0,2000
V6 time >180 mg/dl [%]	12	15,7083	17,8000	5,2000	23,5000	10,4000

Variable	Descriptive Statistics (Dataset_29.01)	
	Upper Quartile	Std.Dev.
V6 weight [kg]	78,0000	12,33564
V6 height [cm]	182,5000	11,40541
V6 BMI [kg/m2]	25,4500	2,64981
V6 HbA1c [%]	6,4000	0,35792
V6 HbA1c [mmol/l]	46,4520	3,91206
V6 fructosamine [umol/l]	333,0000	22,27293
V6 SD DDI [U]	9,2500	2,94220
V6 mean DDI [U]	53,3300	11,77639
V6 DDI [U/kg b.w.]	0,6806	0,10517
V6 mean glucose [mg/dl]	144,8500	8,68658
V6 median glucose [mg/dl]	137,0000	7,71608
V6 SD glucose [mg/dl]	46,3000	6,84409
V6 CV glucose [%]	32,7253	3,69521
V6 time 70-180 mg/dl [%]	86,4500	5,63173
V6 time 70-140 mg/dl [%]	64,1000	7,62118
V6 time <70 mg/dl [%]	3,0500	1,50964
V6 time <54 mg/dl [%]	0,5500	0,53908
V6 time >180 mg/dl [%]	19,1000	5,78658

Variable	Descriptive Statistics (Dataset_29.01)				
	Valid N	Mean	Median	Minimum	Maximum
StudyPeriod weight [kg]	12	71,3083	69,4500	49,2000	100,1000
StudyPeriod height [cm]	12	176,0833	177,0000	160,0000	194,0000
StudyPeriod BMI [kg/m2]	12	22,9167	22,9000	19,0000	26,6000
StudyPeriod HbA1c [%]	12	6,2917	6,3000	5,9000	7,1000
StudyPeriod HbA1c [mmol/l]	12	45,2679	45,3590	40,9870	54,1030
StudyPeriod fructosamine [umol/l]	12	314,5833	315,0000	286,0000	353,0000
StudyPeriod SD DDI [U]	12	7,0300	7,0750	3,0700	10,8400
StudyPeriod mean DDI [U]	12	43,5067	42,2300	20,0000	60,9900
StudyPeriod DDI [U/kg b.w.]	12	0,6033	0,5973	0,4065	0,7819
StudyPeriod mean glucose [mg/dl]	12	141,0667	144,9000	125,5000	149,5000
StudyPeriod median glucose [mg/dl]	12	132,5833	136,0000	118,0000	139,0000
StudyPeriod SD glucose [mg/dl]	12	46,9167	47,8500	33,0000	62,7000
StudyPeriod CV glucose [%]	12	33,1094	33,2573	25,9230	42,6821
StudyPeriod time 70-180 mg/dl [%]	12	79,2917	78,2500	69,0000	91,3000
StudyPeriod time 70-140 mg/dl [%]	12	54,3833	51,0000	46,2000	67,7000
StudyPeriod time <70 mg/dl [%]	12	2,2167	1,7500	0,2000	6,2000
StudyPeriod time <54 mg/dl [%]	12	0,5250	0,3500	0,0000	2,1000
StudyPeriod time >180 mg/dl [%]	12	18,4917	20,8500	6,2000	24,8000

Variable	Descriptive Statistics (Dataset_29.01)		
	Lower Quartile	Upper Quartile	Std.Dev.
StudyPeriod weight [kg]	65,0000	78,0000	12,33564
StudyPeriod height [cm]	167,0000	182,5000	11,40541
StudyPeriod BMI [kg/m2]	20,8500	25,4500	2,64981
StudyPeriod HbA1c [%]	6,0000	6,4000	0,35792
StudyPeriod HbA1c [mmol/l]	42,0800	46,4520	3,91206
StudyPeriod fructosamine [umol/l]	296,0000	333,0000	22,27293
StudyPeriod SD DDI [U]	4,7100	9,3300	2,56641
StudyPeriod mean DDI [U]	37,8900	51,7850	11,57765
StudyPeriod DDI [U/kg b.w.]	0,5493	0,6609	0,09851
StudyPeriod mean glucose [mg/dl]	135,2500	146,6500	8,43492
StudyPeriod median glucose [mg/dl]	126,5000	139,0000	7,27959
StudyPeriod SD glucose [mg/dl]	41,1500	51,4500	8,11741
StudyPeriod CV glucose [%]	30,1091	35,0649	4,37171
StudyPeriod time 70-180 mg/dl [%]	75,0500	83,6500	6,35159
StudyPeriod time 70-140 mg/dl [%]	48,8000	61,1000	7,49240
StudyPeriod time <70 mg/dl [%]	1,5000	2,8500	1,57008
StudyPeriod time <54 mg/dl [%]	0,1500	0,7000	0,58016
StudyPeriod time >180 mg/dl [%]	14,7500	23,0500	6,08597

Variable	Tests of Normality (Dataset_29.01)				
	N	max D	K-S p	W	p
V0 weight [kg]	12	0,201247	p > .20	0,899289	0,155302
V0 height [cm]	12	0,176052	p > .20	0,921819	0,301342

Variable	Tests of Normality (Dataset_29.01)				
	N	max D	K-S p	W	p
V0 BMI [kg/m2]	12	0,228050	p > .20	0,874082	0,073643
V0 HbA1c [%]	12	0,217337	p > .20	0,878714	0,084386
V0 HbA1c [mmol/l]	12	0,217337	p > .20	0,878714	0,084386
V0 fructosamine [umol/l]	11	0,201172	p > .20	0,884824	0,101075
V0 mean sensor glucose 4 weeks [mg/dl]	12	0,158261	p > .20	0,887648	0,109898
V0 SD sensor glucose 4 weeks[mg/dl]	12	0,124157	p > .20	0,982390	0,991430
V0 CV sensor glucose 4 weeks [%]	12	0,111770	p > .20	0,944184	0,554088
V0 DDI 4 weeks [U]	12	0,202518	p > .20	0,941105	0,512521
V0 DDI 4 weeks [U/kg b.w.]	12	0,192426	p > .20	0,861313	0,050775
V0 time <54 mg/dl 4 weeks [%]	12	0,394677	p < ,05	0,625780	0,000177
V0 time <70 mg/dl 4 weeks [%]	12	0,171846	p > .20	0,897083	0,145444
V0 time 70-180 mg/dl 4 weeks [%]	12	0,210888	p > .20	0,892569	0,127183
V0 time 70-140 mg/dl 4 weeks [%]	12	0,217337	p > .20	0,878714	0,084386
V0 time >180 mg/dl 4 weeks [%]	12	0,201172	p > .20	0,884824	0,101075

Variable	Tests of Normality (Dataset_29.01)				
	N	max D	K-S p	W	p
V3 weight [kg]	12	0,201012	p > .20	0,877786	0,082110
V3 height [cm]	12	0,183272	p > .20	0,930443	0,384834
V3 BMI [kg/m2]	12	0,192129	p > .20	0,939986	0,497886
V3 HbA1c [%]	12	0,194735	p > .20	0,911306	0,221727
V3 HbA1c [mmol/l]	12	0,194735	p > .20	0,911306	0,221727
V3 fructosamine [umol/l]	12	0,217832	p > .20	0,901017	0,163486
V3 SD DDI [U]	12	0,195522	p > .20	0,867181	0,060193
V3 mean DDI [U]	12	0,186224	p > .20	0,928968	0,369297
V3 DDI [U/kg b.w.]	12	0,197014	p > .20	0,949236	0,625850
V3 mean glucose [mg/dl]	12	0,162660	p > .20	0,921811	0,301270
V3 median glucose [mg/dl]	12	0,167066	p > .20	0,925333	0,333252
V3 SD glucose [mg/dl]	12	0,122849	p > .20	0,977867	0,973709
V3 CV glucose [%]	12	0,154414	p > .20	0,969909	0,909848
V3 time 70-180 mg/dl [%]	12	0,199459	p > .20	0,943299	0,541947
V3 time 70-140 mg/dl [%]	12	0,147409	p > .20	0,934063	0,425176
V3 time <70 mg/dl [%]	12	0,242755	p > .20	0,789687	0,007169
V3 time <54 mg/dl [%]	12	0,333333	p < ,15	0,652284	0,000304
V3 time >180 mg/dl [%]	12	0,210359	p > .20	0,888313	0,112085

Variable	Tests of Normality (Dataset_29.01)				
	N	max D	K-S p	W	p
V4 weight [kg]	12	0,177788	p > .20	0,905851	0,188705
V4 height [cm]	12	0,183272	p > .20	0,930443	0,384834
V4 BMI [kg/m2]	12	0,156271	p > .20	0,935687	0,444290
V4 HbA1c [%]	12	0,179955	p > .20	0,946267	0,583216
V4 HbA1c [mmol/l]	12	0,179955	p > .20	0,946267	0,583216

Variable	Tests of Normality (Dataset_29.01)				
	N	max D	K-S p	W	p
V4 fructosamine [umol/l]	12	0,134201	p > .20	0,958719	0,765394
V4 SD DDI [U]	12	0,166711	p > .20	0,933335	0,416802
V4 mean DDI [U]	12	0,180120	p > .20	0,920262	0,288089
V4 DDI [U/kg b.w.]	12	0,224395	p > .20	0,920639	0,291248
V4 mean glucose [mg/dl]	12	0,174152	p > .20	0,923882	0,319731
V4 median glucose [mg/dl]	12	0,115321	p > .20	0,972008	0,930662
V4 SD glucose [mg/dl]	12	0,222550	p > .20	0,939878	0,496499
V4 CV glucose [%]	12	0,196043	p > .20	0,948977	0,622083
V4 time 70-180 mg/dl [%]	12	0,120091	p > .20	0,981540	0,989043
V4 time 70-140 mg/dl [%]	12	0,195976	p > .20	0,963887	0,837541
V4 time <70 mg/dl [%]	12	0,177376	p > .20	0,921577	0,299246
V4 time <54 mg/dl [%]	12	0,271706	p > .20	0,819831	0,015887
V4 time >180 mg/dl [%]	12	0,152013	p > .20	0,952365	0,671775

Variable	Tests of Normality (Dataset_29.01)				
	N	max D	K-S p	W	p
V5 weight [kg]	12	0,169980	p > .20	0,919026	0,277949
V5 height [cm]	12	0,183272	p > .20	0,930443	0,384834
V5 BMI [kg/m2]	12	0,168861	p > .20	0,932001	0,401810
V5 HbA1c [%]	12	0,187431	p > .20	0,972777	0,937609
V5 HbA1c [mmol/l]	12	0,187431	p > .20	0,972777	0,937609
V5 fructosamine [umol/l]	11	0,173247	p > .20	0,940443	0,394834
V5 SD DDI [U]	12	0,194716	p > .20	0,921775	0,300956
V5 mean DDI mean [U]	12	0,162246	p > .20	0,968230	0,891406
V5 DDI [U/kg b.w.]	12	0,108767	p > .20	0,980139	0,984197
V5 mean glucose [mg/dl]	12	0,167428	p > .20	0,906887	0,194586
V5 median glucose [mg/dl]	12	0,166171	p > .20	0,921123	0,295354
V5 SD glucose [mg/dl]	12	0,132577	p > .20	0,972820	0,937983
V5 CV glucose [%]	12	0,129339	p > .20	0,955474	0,717807
V5 time 70-180 mg/dl [%]	12	0,166536	p > .20	0,928269	0,362125
V5 time 70-140 mg/dl [%]	12	0,162256	p > .20	0,969230	0,894406
V5 time <70 mg/dl [%]	12	0,251581	p > .20	0,709615	0,001045
V5 time <54 mg/dl [%]	12	0,303909	p < ,20	0,633414	0,000207
V5 time >180 mg/dl [%]	12	0,178766	p > .20	0,867649	0,061020

Variable	Tests of Normality (Dataset_29.01)				
	N	max D	K-S p	W	p
V6 weight [kg]	12	0,191182	p > .20	0,925911	0,338774
V6 height [cm]	12	0,183272	p > .20	0,930443	0,384834
V6 BMI [kg/m2]	12	0,158124	p > .20	0,934570	0,431080
V6 HbA1c [%]	12	0,214402	p > .20	0,892890	0,128400
V6 HbA1c [mmol/l]	12	0,214402	p > .20	0,892890	0,128400
V6 fructosamine [umol/l]	12	0,188395	p > .20	0,937596	0,467574

Variable	Tests of Normality (Dataset_29.01)				
	N	max D	K-S p	W	p
V6 SD DDI [U]	12	0,176869	p > .20	0,901070	0,163745
V6 mean DDI [U]	12	0,130890	p > .20	0,971341	0,924333
V6 DDI [U/kg b.w.]	12	0,141964	p > .20	0,982292	0,991176
V6 mean glucose [mg/dl]	12	0,173262	p > .20	0,894015	0,132768
V6 median glucose [mg/dl]	12	0,201434	p > .20	0,875155	0,075999
V6 SD glucose [mg/dl]	12	0,184443	p > .20	0,952399	0,672276
V6 CV glucose [%]	12	0,209066	p > .20	0,904230	0,179849
V6 time 70-180 mg/dl [%]	12	0,146818	p > .20	0,977104	0,969413
V6 time 70-140 mg/dl [%]	12	0,175400	p > .20	0,952033	0,666874
V6 time <70 mg/dl [%]	12	0,133848	p > .20	0,946532	0,586972
V6 time <54 mg/dl [%]	12	0,311426	p < ,20	0,729218	0,001635
V6 time >180 mg/dl [%]	12	0,185045	p > .20	0,930326	0,383580

Variable	Tests of Normality (Dataset_29.01)				
	N	max D	K-S p	W	p
StudyPeriod weight [kg]	12	0,191182	p > .20	0,925911	0,338774
StudyPeriod height [cm]	12	0,183272	p > .20	0,930443	0,384834
StudyPeriod BMI [kg/m2]	12	0,158124	p > .20	0,934570	0,431080
StudyPeriod HbA1c [%]	12	0,214402	p > .20	0,892890	0,128400
StudyPeriod HbA1c [mmol/l]	12	0,214402	p > .20	0,892890	0,128400
StudyPeriod fructosamine [umol/l]	12	0,188395	p > .20	0,937596	0,467574
StudyPeriod SD DDI [U]	12	0,143011	p > .20	0,948557	0,615993
StudyPeriod mean DDI [U]	12	0,150017	p > .20	0,955860	0,723506
StudyPeriod DDI [U/kg b.w.]	12	0,129307	p > .20	0,987204	0,998676
StudyPeriod mean glucose [mg/dl]	12	0,188395	p > .20	0,937596	0,467574
StudyPeriod median glucose [mg/dl]	12	0,188395	p > .20	0,937596	0,467574
StudyPeriod SD glucose [mg/dl]	12	0,101275	p > .20	0,988356	0,999288
StudyPeriod CV glucose [%]	12	0,137701	p > .20	0,965040	0,852589
StudyPeriod time 70-180 mg/dl [%]	12	0,180737	p > .20	0,941720	0,520667
StudyPeriod time 70-140 mg/dl [%]	12	0,196283	p > .20	0,874460	0,074465
StudyPeriod time <70 mg/dl [%]	12	0,231096	p > .20	0,851882	0,038733
StudyPeriod time <54 mg/dl [%]	12	0,198571	p > .20	0,787377	0,006756
StudyPeriod time >180 mg/dl [%]	12	0,183272	p > .20	0,930443	0,384834

Effect	Repeated Measures Analysis of Variance with Effect Sizes and Powers (Dataset_29.01)<br>Sigma-restricted parameterization<br>Effective hypothesis decomposition					
	SS	Degr. of Freedom	MS	F	p	Partial eta-squared
Intercept	180554,2	1	180554,2	409,0054	0,000000	0,973810
Error	4855,9	11	441,4			
WEIGHT	4,3	2	2,2	1,2266	0,312583	0,100321
Error	39,0	22	1,8			

Effect	Repeated Measures Analysis of Variance with Effect Sizes and Powers (Dataset_29.01) Sigma-restricted parameterization Effective hypothesis decomposition	
	Non-centrality	Observed power (alpha=0,05)
Intercept	409,0054	1,000000
Error		
WEIGHT	2,4532	0,239173
Error		

Cell No.	Tukey HSD test; variable DV_1 (Dataset_29.01) Homogenous Groups, alpha = ,05000 Error: Within MS = 1,7707, df = 22,000		
	WEIGHT	DV_1 Mean	1
1	V0 weight [kg]	70,53333	****
2	V3 weight [kg]	70,61667	****
3	StudyPeriod weight [kg]	71,30833	****

Effect	Mauchley Sphericity Test (Dataset_29.01) Sigma-restricted parameterization Effective hypothesis decomposition			
	W	Chi-Sqr.	df	p
WEIGHT	0,614356	4,871804	2	0,087519

Effect	Repeated Measures Analysis of Variance with Effect Sizes and Powers (Dataset_29.01) Sigma-restricted parameterization Effective hypothesis decomposition					
	SS	Degr. of Freedom	MS	F	p	Partial eta-squared
Intercept	18700,56	1	18700,56	979,7049	0,000000	0,988897
Error	209,97	11	19,09			
BMI	0,30	2	0,15	0,7248	0,495637	0,061817
Error	4,58	22	0,21			

Effect	Repeated Measures Analysis of Variance with Effect Sizes and Powers (Dataset_29.01) Sigma-restricted parameterization Effective hypothesis decomposition	
	Non-centrality	Observed power (alpha=0,05)
Intercept	979,7049	1,000000
Error		
BMI	1,4496	0,156827

Effect	Repeated Measures Analysis of Variance with Effect Sizes and Powers (Dataset_29.01)<br>Sigma-restricted parameterization<br>Effective hypothesis decomposition	
	Non-centrality	Observed power (alpha=0,05)
Error		

Cell No.	Tukey HSD test; variable DV_1 (Dataset_29.01)<br>Homogenous Groups, alpha = ,05000<br>Error: Within MS = ,20811, df = 22,000		
	BMI	DV_1 Mean	1
2	V3 BMI [kg/m2]	22,70000	****
1	V0 BMI [kg/m2]	22,75833	****
3	StudyPeriod BMI [kg/m2]	22,91667	****

Effect	Mauchley Sphericity Test (Dataset_29.01)<br>Sigma-restricted parameterization<br>Effective hypothesis decomposition			
	W	Chi-Sqr.	df	p
BMI	0,579625	5,453738	2	0,065424

Effect	Repeated Measures Analysis of Variance with Effect Sizes and Powers (Dataset_29.01)<br>Sigma-restricted parameterization<br>Effective hypothesis decomposition					
	SS	Degr. of Freedom	MS	F	p	Partial eta-squared
Intercept	1551,047	1	1551,047	2777,366	0,000000	0,996055
Error	6,143	11	0,558			
HBA1C%	1,816	2	0,908	23,650	0,000003	0,682540
Error	0,844	22	0,038			

Effect	Repeated Measures Analysis of Variance with Effect Sizes and Powers (Dataset_29.01)<br>Sigma-restricted parameterization<br>Effective hypothesis decomposition	
	Non-centrality	Observed power (alpha=0,05)
Intercept	2777,366	1,000000
Error		
HBA1C%	47,300	0,999976
Error		

Cell No.	Tukey HSD test; variable DV_1 (Dataset_29.01)<br>Homogenous Groups, alpha = ,05000<br>Error: Within MS = ,03838, df = 22,000				
	HBA1C%	DV_1 Mean	1	2	3
3	StudyPeriod HbA1c [%]	6,291667	****		
2	V3 HbA1c [%]	6,558333		****	
1	V0 HbA1c [%]	6,841667			****

Effect	Mauchley Sphericity Test (Dataset_29.01)<br>Sigma-restricted parameterization<br>Effective hypothesis decomposition			
	W	Chi-Sqr.	df	p
HBA1C%	0,797971	2,256831	2	0,323545

Effect	Repeated Measures Analysis of Variance with Effect Sizes and Powers (Dataset_29.01)<br>Sigma-restricted parameterization<br>Effective hypothesis decomposition					
	SS	Degr. of Freedom	MS	F	p	Partial eta-squared
Intercept	83787,00	1	83787,00	1255,870	0,000000	0,991317
Error	733,88	11	66,72			
HBA1C	216,90	2	108,45	23,650	0,000003	0,682540
Error	100,88	22	4,59			

Effect	Repeated Measures Analysis of Variance with Effect Sizes and Powers (Dataset_29.01)<br>Sigma-restricted parameterization<br>Effective hypothesis decomposition	
	Non-centrality	Observed power (alpha=0,05)
Intercept	1255,870	1,000000
Error		
HBA1C	47,300	0,999976
Error		

Cell No.	Tukey HSD test; variable DV_1 (Dataset_29.01)<br>Homogenous Groups, alpha = ,05000<br>Error: Within MS = 4,5855, df = 22,000				
	HBA1C	DV_1 Mean	1	2	3
3	StudyPeriod HbA1c [mmol/l]	45,26792	****		
2	V3 HbA1c [mmol/l]	48,18258		****	

Cell No.	Tukey HSD test; variable DV_1 (Dataset_29.01)<br>Homogenous Groups, alpha = ,05000<br>Error: Within MS = 4,5855, df = 22,000				
	HBA1C	DV_1<br>Mean	1	2	3
1	V0 HbA1c [mmol/l]	51,27942			****

Effect	Mauchley Sphericity Test (Dataset_29.01)<br>Sigma-restricted parameterization<br>Effective hypothesis decomposition			
	W	Chi-Sqr.	df	p
HBA1C	0,797971	2,256831	2	0,323545

Effect	Repeated Measures Analysis of Variance with Effect Sizes and Powers (Dataset_29.01)<br>Sigma-restricted parameterization<br>Effective hypothesis decomposition					
	SS	Degr. of<br>Freedom	MS	F	p	Partial eta-squared
Intercept	3628102	1	3628102	3146,646	0,000000	0,996832
Error	11530	10	1153			
FRUCTO	6708	2	3354	7,198	0,004417	0,418551
Error	9318	20	466			

Effect	Repeated Measures Analysis of Variance with Effect Sizes and Powers (Dataset_29.01)<br>Sigma-restricted parameterization<br>Effective hypothesis decomposition	
	Non-centrality	Observed power (alpha=0,05)
Intercept	3146,646	1,000000
Error		
FRUCTO	14,397	0,891953
Error		

Cell No.	Tukey HSD test; variable DV_1 (Dataset_29.01)<br>Homogenous Groups, alpha = ,05000<br>Error: Within MS = 465,92, df = 20,000			
	FRUCTO	DV_1<br>Mean	1	2
3	StudyPeriod fructosamine [umol/l]	317,1818	****	
2	V3 fructosamine [umol/l]	326,5455	****	
1	V0 fructosamine [umol/l]	351,0000		****

Effect	Mauchley Sphericity Test (Dataset_29.01)<br>Sigma-restricted parameterization<br>Effective hypothesis decomposition			
	W	Chi-Sqr.	df	p
FRUCTO	0,668016	3,630990	2	0,162757

Effect	Repeated Measures Analysis of Variance with Effect Sizes and Powers (Dataset_29.01)<br>Sigma-restricted parameterization<br>Effective hypothesis decomposition					
	SS	Degr. of Freedom	MS	F	p	Partial eta-squared
Intercept	777042,3	1	777042,3	1791,915	0,000000	0,993899
Error	4770,0	11	433,6			
MEANGLU	908,6	2	454,3	8,042	0,002391	0,422326
Error	1242,8	22	56,5			

Effect	Repeated Measures Analysis of Variance with Effect Sizes and Powers (Dataset_29.01)<br>Sigma-restricted parameterization<br>Effective hypothesis decomposition	
	Non-centrality	Observed power (alpha=0,05)
Intercept	1791,915	1,000000
Error		
MEANGLU	16,084	0,927458
Error		

Cell No.	Tukey HSD test; variable DV_1 (Dataset_29.01)<br>Homogenous Groups, alpha = ,05000<br>Error: Within MS = 56,492, df = 22,000			
	MEANGLU	DV_1 Mean	1	2
3	StudyPeriod mean glucose [mg/dl]	141,0667	****	
2	V3 mean glucose [mg/dl]	146,3500	****	****
1	V0 mean sensor glucose 4 weeks [mg/dl]	153,3333		****

Effect	Mauchley Sphericity Test (Dataset_29.01)<br>Sigma-restricted parameterization<br>Effective hypothesis decomposition			
	W	Chi-Sqr.	df	p
MEANGLU	0,778692	2,501397	2	0,286305

Effect	Repeated Measures Analysis of Variance with Effect Sizes and Powers (Dataset_29.01)<br>Sigma-restricted parameterization<br>Effective hypothesis decomposition					
	SS	Degr. of Freedom	MS	F	p	Partial eta-squared
Intercept	91314,77	1	91314,77	361,2113	0,000000	0,970447
Error	2780,82	11	252,80			
SDGLU	394,85	2	197,43	6,4951	0,006071	0,371251
Error	668,72	22	30,40			

Effect	Repeated Measures Analysis of Variance with Effect Sizes and Powers (Dataset_29.01)<br>Sigma-restricted parameterization<br>Effective hypothesis decomposition	
	Non-centrality	Observed power (alpha=0,05)
Intercept	361,2113	1,000000
Error		
SDGLU	12,9901	0,862185
Error		

Cell No.	Tukey HSD test; variable DV_1 (Dataset_29.01)<br>Homogenous Groups, alpha = ,05000<br>Error: Within MS = 30,396, df = 22,000			
	SDGLU	DV_1 Mean	1	2
3	StudyPeriod SD glucose [mg/dl]	46,91667	****	
2	V3 SD glucose [mg/dl]	49,34167	****	****
1	V0 SD sensor glucose 4 weeks[mg/dl]	54,83333		****

Effect	Mauchley Sphericity Test (Dataset_29.01)<br>Sigma-restricted parameterization<br>Effective hypothesis decomposition			
	W	Chi-Sqr.	df	p
SDGLU	0,847910	1,649807	2	0,438277

Effect	Repeated Measures Analysis of Variance with Effect Sizes and Powers (Dataset_29.01)<br>Sigma-restricted parameterization<br>Effective hypothesis decomposition					
	SS	Degr. of Freedom	MS	F	p	Partial eta-squared
Intercept	41840,26	1	41840,26	645,1387	0,000000	0,983235
Error	713,40	11	64,85			
CV	46,51	2	23,25	2,6025	0,096722	0,191323
Error	196,57	22	8,94			

Effect	Repeated Measures Analysis of Variance with Effect Sizes and Powers (Dataset_29.01)<br>Sigma-restricted parameterization<br>Effective hypothesis decomposition	
	Non-centrality	Observed power (alpha=0,05)
Intercept	645,1387	1,000000
Error		
CV	5,2049	0,463817
Error		

Effect	Mauchley Sphericity Test (Dataset_29.01)<br>Sigma-restricted parameterization<br>Effective hypothesis decomposition			
	W	Chi-Sqr.	df	p
CV	0,876673	1,316211	2	0,517831

Effect	Repeated Measures Analysis of Variance with Effect Sizes and Powers (Dataset_29.01)<br>Sigma-restricted parameterization<br>Effective hypothesis decomposition					
	SS	Degr. of Freedom	MS	F	p	Partial eta-squared
Intercept	13,16830	1	13,16830	592,7488	0,000000	0,981781
Error	0,24437	11	0,02222			
DDI	0,00297	2	0,00148	1,0529	0,365845	0,087359
Error	0,03100	22	0,00141			

Effect	Repeated Measures Analysis of Variance with Effect Sizes and Powers (Dataset_29.01)<br>Sigma-restricted parameterization<br>Effective hypothesis decomposition	
	Non-centrality	Observed power (alpha=0,05)
Intercept	592,7488	1,000000
Error		
DDI	2,1059	0,210290
Error		

Cell No.	Tukey HSD test; variable DV_1 (Dataset_29.01)<br>Homogenous Groups, alpha = ,05000<br>Error: Within MS = ,00141, df = 22,000		
	DDI	DV_1<br>Mean	1
2	V3 DDI [U/kg b.w.]	0,594520	****
3	StudyPeriod DDI [U/kg b.w.]	0,603284	****
1	V0 DDI 4 weeks [U/kg b.w.]	0,616604	****

Effect	Mauchley Sphericity Test (Dataset_29.01)<br>Sigma-restricted parameterization<br>Effective hypothesis decomposition			
	W	Chi-Sqr.	df	p
DDI	0,854988	1,566680	2	0,456877

Variable	Friedman ANOVA and Kendall Coeff. of Concordance (Dataset_29.01)<br>ANOVA Chi Sqr. (N = 12, df = 2) = 1,086957 p = ,58073<br>Coeff. of Concordance = ,04529 Aver. rank r = -,0415			
	Average Rank	Sum of Ranks	Mean	Std.Dev.
V0 time <54 mg/dl 4 weeks [%]	1,791667	21,50000	0,910833	1,565639
V3 time <54 mg/dl [%]	2,000000	24,00000	0,700000	1,149704
StudyPeriod time <54 mg/dl [%]	2,208333	26,50000	0,525000	0,580165

Variable	Friedman ANOVA and Kendall Coeff. of Concordance (Dataset_29.01)<br>ANOVA Chi Sqr. (N = 12, df = 2) = ,2978723 p = ,86162<br>Coeff. of Concordance = ,01241 Aver. rank r = -,0774			
	Average Rank	Sum of Ranks	Mean	Std.Dev.
V0 time <70 mg/dl 4 weeks [%]	2,041667	24,50000	3,318333	2,950044
V3 time <70 mg/dl [%]	1,875000	22,50000	2,608333	2,583324
StudyPeriod time <70 mg/dl [%]	2,083333	25,00000	2,216667	1,570080

Effect	Repeated Measures Analysis of Variance with Effect Sizes and Powers (Dataset_29.01)<br>Sigma-restricted parameterization<br>Effective hypothesis decomposition					
	SS	Degr. of Freedom	MS	F	p	Partial eta-squared
Intercept	196754,3	1	196754,3	786,3221	0,000000	0,986204
Error	2752,4	11	250,2			
70-180	764,0	2	382,0	12,3225	0,000257	0,528353
Error	682,0	22	31,0			

Effect	Repeated Measures Analysis of Variance with Effect Sizes and Powers (Dataset_29.01)<br>Sigma-restricted parameterization<br>Effective hypothesis decomposition	
	Non-centrality	Observed power (alpha=0,05)
Intercept	786,3221	1,000000
Error		
70-180	24,6450	0,989889
Error		

Cell No.	Tukey HSD test; variable DV_1 (Dataset_29.01)<br>Homogenous Groups, alpha = ,05000<br>Error: Within MS = 31,002, df = 22,000			
	70-180	DV_1 Mean	1	2
1	V0 time 70-180 mg/dl 4 weeks [%]	68,04333		****
2	V3 time 70-180 mg/dl [%]	74,45000	****	
3	StudyPeriod time 70-180 mg/dl [%]	79,29167	****	

Effect	Mauchley Sphericity Test (Dataset_29.01)<br>Sigma-restricted parameterization<br>Effective hypothesis decomposition			
	W	Chi-Sqr.	df	p
70-180	0,842137	1,718128	2	0,423558

Effect	Repeated Measures Analysis of Variance with Effect Sizes and Powers (Dataset_29.01)<br>Sigma-restricted parameterization<br>Effective hypothesis decomposition					
	SS	Degr. of Freedom	MS	F	p	Partial eta-squared
Intercept	86378,19	1	86378,19	283,6858	0,000000	0,962672
Error	3349,34	11	304,49			

Effect	Repeated Measures Analysis of Variance with Effect Sizes and Powers (Dataset_29.01)<br>Sigma-restricted parameterization<br>Effective hypothesis decomposition					
	SS	Degr. of Freedom	MS	F	p	Partial eta-squared
70-140	725,45	2	362,72	12,9369	0,000193	0,540458
Error	616,84	22	28,04			

Effect	Repeated Measures Analysis of Variance with Effect Sizes and Powers (Dataset_29.01)<br>Sigma-restricted parameterization<br>Effective hypothesis decomposition	
	Non-centrality	Observed power (alpha=0,05)
Intercept	283,6858	1,000000
Error		
70-140	25,8737	0,992519
Error		

Cell No.	Tukey HSD test; variable DV_1 (Dataset_29.01)<br>Homogenous Groups, alpha = ,05000<br>Error: Within MS = 28,038, df = 22,000			
	70-140	DV_1 Mean	1	2
1	V0 time 70-140 mg/dl 4 weeks [%]	43,39250		****
2	V3 time 70-140 mg/dl [%]	49,17500	****	
3	StudyPeriod time 70-140 mg/dl [%]	54,38333	****	

Effect	Mauchley Sphericity Test (Dataset_29.01)<br>Sigma-restricted parameterization<br>Effective hypothesis decomposition			
	W	Chi-Sqr.	df	p
70-140	0,776739	2,526515	2	0,282731

Effect	Repeated Measures Analysis of Variance with Effect Sizes and Powers (Dataset_29.01)<br>Sigma-restricted parameterization<br>Effective hypothesis decomposition					
	SS	Degr. of Freedom	MS	F	p	Partial eta-squared
Intercept	19626,14	1	19626,14	87,15877	0,000001	0,887937
Error	2476,95	11	225,18			
180	621,09	2	310,55	10,96831	0,000496	0,499279
Error	622,89	22	28,31			

Effect	Repeated Measures Analysis of Variance with Effect Sizes and Powers (Dataset_29.01)<br>Sigma-restricted parameterization<br>Effective hypothesis decomposition	
	Non-centrality	Observed power (alpha=0,05)
Intercept	87,15877	1,000000
Error		
180	21,93663	0,980644
Error		

Cell No.	Tukey HSD test; variable DV_1 (Dataset_29.01)<br>Homogenous Groups, alpha = ,05000<br>Error: Within MS = 28,313, df = 22,000			
	180	DV_1 Mean	1	2
3	StudyPeriod time >180 mg/dl [%]	18,49167	****	
2	V3 time >180 mg/dl [%]	22,91667	****	
1	V0 time >180 mg/dl 4 weeks [%]	28,63833		****

Effect	Mauchley Sphericity Test (Dataset_29.01)<br>Sigma-restricted parameterization<br>Effective hypothesis decomposition			
	W	Chi-Sqr.	df	p
180	0,849571	1,630232	2	0,442588

Effect	Repeated Measures Analysis of Variance with Effect Sizes and Powers (Dataset_29.01)<br>Sigma-restricted parameterization<br>Effective hypothesis decomposition					
	SS	Degr. of Freedom	MS	F	p	Partial eta-squared
Intercept	943798,4	1	943798,4	3195,798	0,000000	0,996570
Error	3248,6	11	295,3			
MEANGLU	1868,7	3	622,9	17,798	0,000000	0,618035
Error	1154,9	33	35,0			

Effect	Repeated Measures Analysis of Variance with Effect Sizes and Powers (Dataset_29.01)<br>Sigma-restricted parameterization<br>Effective hypothesis decomposition	
	Non-centrality	Observed power (alpha=0,05)
Intercept	3195,798	1,000000
Error		
MEANGLU	53,395	0,999991
Error		

Cell No.	Tukey HSD test; variable DV_1 (Dataset_29.01)<br>Homogenous Groups, alpha = ,05000<br>Error: Within MS = 34,997, df = 33,000			
	MEANGLU	DV_1<br>Mean	1	2
4	V6 median glucose [mg/dl]	129,9167		****
3	V5 mean glucose [mg/dl]	141,0417	****	
2	V4 mean glucose [mg/dl]	143,5833	****	
1	V3 mean glucose [mg/dl]	146,3500	****	

Effect	Mauchley Sphericity Test (Dataset_29.01)<br>Sigma-restricted parameterization<br>Effective hypothesis decomposition			
	W	Chi-Sqr.	df	p
MEANGLU	0,306126	11,50878	5	0,042175

Effect	Repeated Measures Analysis of Variance with Effect Sizes and Powers (Dataset_29.01)<br>Sigma-restricted parameterization<br>Effective hypothesis decomposition					
	SS	Degr. of<br>Freedom	MS	F	p	Partial eta-squared
Intercept	106257,7	1	106257,7	369,7025	0,000000	0,971106
Error	3161,6	11	287,4			
SD	171,4	3	57,1	2,7595	0,057690	0,200551
Error	683,1	33	20,7			

Effect	Repeated Measures Analysis of Variance with Effect Sizes and Powers (Dataset_29.01)<br>Sigma-restricted parameterization<br>Effective hypothesis decomposition	
	Non-centrality	Observed power (alpha=0,05)
Intercept	369,7025	1,000000
Error		
SD	8,2784	0,613437
Error		

Cell No.	Tukey HSD test; variable DV_1 (Dataset_29.01) Homogenous Groups, alpha = ,05000 Error: Within MS = 20,701, df = 33,000			
	SD	DV_1 Mean	1	2
4	V6 SD glucose [mg/dl]	44,08333	****	
2	V4 SD glucose [mg/dl]	47,37500	****	****
3	V5 SD glucose [mg/dl]	47,40000	****	****
1	V3 SD glucose [mg/dl]	49,34167		****

Effect	Mauchley Sphericity Test (Dataset_29.01) Sigma-restricted parameterization Effective hypothesis decomposition			
	W	Chi-Sqr.	df	p
SD	0,214567	14,96380	5	0,010518

Effect	Adjusted Univariate Tests for Repeated Measure: DV_1 (Dataset_29.01) Sigma-restricted parameterization Effective hypothesis decomposition						
	Degr. of Freedom	F	p	G-G Epsilon	G-G Adj. df1	G-G Adj. df2	G-G Adj. p
SD	3	2,759474	0,057690	0,556846	1,670538	18,37592	0,096990
Error	33						

Effect	Adjusted Univariate Tests for Repeated Measure: DV_1 (Dataset_29.01) Sigma-restricted parameterization Effective hypothesis decomposition						
	H-F Epsilon	H-F Adj. df1	H-F Adj. df2	H-F Adj. p	Lowr.Bnd Epsilon	Lowr.Bnd Adj. df1	Lowr.Bnd Adj. df2
SD	0,644784	1,934351	21,27786	0,087475	0,333333	1,000000	11,00000
Error							

Effect	Adjusted Univariate Tests for Repeated Measure: DV_1 (Dataset_29.01) Sigma-restricted parameterization Effective hypothesis decomposition
	Lowr.Bnd Adj. p
SD	0,124885
Error	

Effect	Repeated Measures Analysis of Variance with Effect Sizes and Powers (Dataset_29.01) Sigma-restricted parameterization Effective hypothesis decomposition					
	SS	Degr. of Freedom	MS	F	p	Partial eta-squared
Intercept	52007,62	1	52007,62	635,2750	0,000000	0,982979
Error	900,53	11	81,87			
CV	20,47	3	6,82	1,0727	0,374047	0,088852
Error	209,88	33	6,36			

Effect	Repeated Measures Analysis of Variance with Effect Sizes and Powers (Dataset_29.01) Sigma-restricted parameterization Effective hypothesis decomposition	
	Non-centrality	Observed power (alpha=0,05)
Intercept	635,2750	1,000000
Error		
CV	3,2181	0,263283
Error		

Cell No.	Tukey HSD test; variable DV_1 (Dataset_29.01) Homogenous Groups, alpha = ,05000 Error: Within MS = 6,3601, df = 33,000		
	CV	DV_1 Mean	1
4	V6 CV glucose [%]	31,92665	****
2	V4 CV glucose [%]	32,73109	****

Cell No.	Tukey HSD test; variable DV_1 (Dataset_29.01)<br>Homogenous Groups, alpha = ,05000<br>Error: Within MS = 6,3601, df = 33,000		
	CV	DV_1 Mean	1
1	V3 CV glucose [%]	33,48048	****
3	V5 CV glucose [%]	33,52753	****

Effect	Mauchley Sphericity Test (Dataset_29.01)<br>Sigma-restricted parameterization<br>Effective hypothesis decomposition			
	W	Chi-Sqr.	df	p
CV	0,475252	7,232468	5	0,203917

Variable	Friedman ANOVA and Kendall Coeff. of Concordance (Dataset_29.01)<br>ANOVA Chi Sqr. (N = 12, df = 3) = ,1926606 p = ,97877<br>Coeff. of Concordance = ,00535 Aver. rank r = -,0851			
	Average Rank	Sum of Ranks	Mean	Std.Dev.
V3 time <54 mg/dl [%]	2,500000	30,00000	0,700000	1,149704
V4 time <54 mg/dl [%]	2,416667	29,00000	0,508333	0,548483
V5 time <54 mg/dl [%]	2,458333	29,50000	0,500000	0,698049
V6 time <54 mg/dl [%]	2,625000	31,50000	0,483333	0,539079

Variable	Friedman ANOVA and Kendall Coeff. of Concordance (Dataset_29.01)<br>ANOVA Chi Sqr. (N = 12, df = 3) = ,7118644 p = ,87041<br>Coeff. of Concordance = ,01977 Aver. rank r = -,0693			
	Average Rank	Sum of Ranks	Mean	Std.Dev.
V3 time <70 mg/dl [%]	2,500000	30,00000	2,608333	2,583324
V4 time <70 mg/dl [%]	2,250000	27,00000	1,916667	1,339493
V5 time <70 mg/dl [%]	2,666667	32,00000	2,308333	2,103442
V6 time <70 mg/dl [%]	2,583333	31,00000	2,191667	1,509641

Effect	Repeated Measures Analysis of Variance with Effect Sizes and Powers (Dataset_29.01)<br>Sigma-restricted parameterization<br>Effective hypothesis decomposition					
	SS	Degr. of Freedom	MS	F	p	Partial eta-squared
Intercept	297265,6	1	297265,6	1517,979	0,000000	0,992806
Error	2154,1	11	195,8			
70-180	363,8	3	121,3	6,755	0,001120	0,380471
Error	592,4	33	18,0			

Effect	Repeated Measures Analysis of Variance with Effect Sizes and Powers (Dataset_29.01)<br>Sigma-restricted parameterization<br>Effective hypothesis decomposition	
	Non-centrality	Observed power (alpha=0,05)
Intercept	1517,979	1,000000
Error		
70-180	20,266	0,958732
Error		

Cell No.	Tukey HSD test; variable DV_1 (Dataset_29.01)<br>Homogenous Groups, alpha = ,05000<br>Error: Within MS = 17,951, df = 33,000			
	70-180	DV_1 Mean	1	2
1	V3 time 70-180 mg/dl [%]	74,45000		****
2	V4 time 70-180 mg/dl [%]	78,70000	****	****
3	V5 time 70-180 mg/dl [%]	79,53333	****	
4	V6 time 70-180 mg/dl [%]	82,10000	****	

Effect	Mauchley Sphericity Test (Dataset_29.01)<br>Sigma-restricted parameterization<br>Effective hypothesis decomposition			
	W	Chi-Sqr.	df	p
70-180	0,235695	14,05072	5	0,015290

Effect	Adjusted Univariate Tests for Repeated Measure: DV_1 (Dataset_29.01)<br>Sigma-restricted parameterization<br>Effective hypothesis decomposition						
	Degr. of Freedom	F	p	G-G Epsilon	G-G Adj. df1	G-G Adj. df2	G-G Adj. p
70-180	3	6,755422	0,001120	0,537010	1,611031	17,72134	0,009445

Effect	Adjusted Univariate Tests for Repeated Measure: DV_1 (Dataset_29.01)<br>Sigma-restricted parameterization<br>Effective hypothesis decomposition						
	Degr. of Freedom	F	p	G-G Epsilon	G-G Adj. df1	G-G Adj. df2	G-G Adj. p
Error	33						

Effect	Adjusted Univariate Tests for Repeated Measure: DV_1 (Dataset_29.01)<br>Sigma-restricted parameterization<br>Effective hypothesis decomposition						
	H-F Epsilon	H-F Adj. df1	H-F Adj. df2	H-F Adj. p	Lowr.Bnd Epsilon	Lowr.Bnd Adj. df1	Lowr.Bnd Adj. df2
70-180	0,615345	1,846036	20,30639	0,006550	0,333333	1,000000	11,00000
Error							

Effect	Adjusted Univariate Tests for Repeated Measure: DV_1 (Dataset_29.01)<br>Sigma-restricted parameterization<br>Effective hypothesis decomposition
	Lowr.Bnd Adj. p
70-180	0,024733
Error	

Effect	Repeated Measures Analysis of Variance with Effect Sizes and Powers (Dataset_29.01)<br>Sigma-restricted parameterization<br>Effective hypothesis decomposition					
	SS	Degr. of Freedom	MS	F	p	Partial eta-squared
Intercept	141050,1	1	141050,1	542,1351	0,000000	0,980113
Error	2861,9	11	260,2			
70-140	499,5	3	166,5	6,2847	0,001710	0,363599
Error	874,2	33	26,5			

Effect	Repeated Measures Analysis of Variance with Effect Sizes and Powers (Dataset_29.01)<br>Sigma-restricted parameterization<br>Effective hypothesis decomposition	
	Non-centrality	Observed power (alpha=0,05)
Intercept	542,1351	1,000000
Error		
70-140	18,8541	0,944323

Effect	Repeated Measures Analysis of Variance with Effect Sizes and Powers (Dataset_29.01)<br>Sigma-restricted parameterization<br>Effective hypothesis decomposition	
	Non-centrality	Observed power (alpha=0,05)
Error		

Cell No.	Tukey HSD test; variable DV_1 (Dataset_29.01)<br>Homogenous Groups, alpha = ,05000<br>Error: Within MS = 26,491, df = 33,000			
	70-140	DV_1<br>Mean	1	2
1	V3 time 70-140 mg/dl [%]	49,17500		****
2	V4 time 70-140 mg/dl [%]	54,20000	****	****
3	V5 time 70-140 mg/dl [%]	55,39167	****	
4	V6 time 70-140 mg/dl [%]	58,06667	****	

Effect	Mauchley Sphericity Test (Dataset_29.01)<br>Sigma-restricted parameterization<br>Effective hypothesis decomposition			
	W	Chi-Sqr.	df	p
70-140	0,500698	6,725375	5	0,241879

Effect	Repeated Measures Analysis of Variance with Effect Sizes and Powers (Dataset_29.01)<br>Sigma-restricted parameterization<br>Effective hypothesis decomposition					
	SS	Degr. of<br>Freedom	MS	F	p	Partial eta-squared
Intercept	17404,08	1	17404,08	105,6363	0,000001	0,905690
Error	1812,30	11	164,75			
180	324,29	3	108,10	6,5012	0,001406	0,371471
Error	548,69	33	16,63			

Effect	Repeated Measures Analysis of Variance with Effect Sizes and Powers (Dataset_29.01)<br>Sigma-restricted parameterization<br>Effective hypothesis decomposition	
	Non-centrality	Observed power (alpha=0,05)
Intercept	105,6363	1,000000
Error		
180	19,5036	0,951441
Error		

Cell No.	Tukey HSD test; variable DV_1 (Dataset_29.01)<br>Homogenous Groups, alpha = ,05000<br>Error: Within MS = 16,627, df = 33,000			
	180	DV_1 Mean	1	2
4	V6 time >180 mg/dl [%]	15,70833	****	
3	V5 time >180 mg/dl [%]	18,15833	****	
2	V4 time >180 mg/dl [%]	19,38333	****	****
1	V3 time >180 mg/dl [%]	22,91667		****

Effect	Mauchley Sphericity Test (Dataset_29.01)<br>Sigma-restricted parameterization<br>Effective hypothesis decomposition			
	W	Chi-Sqr.	df	p
180	0,259504	13,11511	5	0,022324

Effect	Adjusted Univariate Tests for Repeated Measure: DV_1 (Dataset_29.01)<br>Sigma-restricted parameterization<br>Effective hypothesis decomposition						
	Degr. of Freedom	F	p	G-G Epsilon	G-G Adj. df1	G-G Adj. df2	G-G Adj. p
180	3	6,501185	0,001406	0,542679	1,628036	17,90840	0,010502
Error	33						

Effect	Adjusted Univariate Tests for Repeated Measure: DV_1 (Dataset_29.01)<br>Sigma-restricted parameterization<br>Effective hypothesis decomposition						
	H-F Epsilon	H-F Adj. df1	H-F Adj. df2	H-F Adj. p	Lowr.Bnd Epsilon	Lowr.Bnd Adj. df1	Lowr.Bnd Adj. df2
180	0,623720	1,871159	20,58275	0,007317	0,333333	1,000000	11,00000
Error							

Effect	Adjusted Univariate Tests for Repeated Measure: DV_1 (Dataset_29.01) Sigma-restricted parameterization Effective hypothesis decomposition
	Lowr.Bnd Adj. p
180	0,027007
Error	

Effect	Repeated Measures Analysis of Variance with Effect Sizes and Powers (Dataset_29.01) Sigma-restricted parameterization Effective hypothesis decomposition					
	SS	Degr. of Freedom	MS	F	p	Partial eta-squared
Intercept	17,29323	1	17,29323	471,3842	0,000000	0,977197
Error	0,40355	11	0,03669			
DDI	0,00098	3	0,00033	0,3031	0,822892	0,026819
Error	0,03563	33	0,00108			

Effect	Repeated Measures Analysis of Variance with Effect Sizes and Powers (Dataset_29.01) Sigma-restricted parameterization Effective hypothesis decomposition	
	Non-centrality	Observed power (alpha=0,05)
Intercept	471,3842	1,000000
Error		
DDI	0,9094	0,102202
Error		

Cell No.	Tukey HSD test; variable DV_1 (Dataset_29.01) Homogenous Groups, alpha = ,05000 Error: Within MS = ,00108, df = 33,000		
	DDI	DV_1 Mean	1
1	V3 DDI [U/kg b.w.]	0,594520	****
4	V6 DDI [U/kg b.w.]	0,598526	****

Cell No.	Tukey HSD test; variable DV_1 (Dataset_29.01) Homogenous Groups, alpha = ,05000 Error: Within MS = ,00108, df = 33,000		
	DDI	DV_1 Mean	1
3	V5 DDI [U/kg b.w.]	0,600867	****
2	V4 DDI [U/kg b.w.]	0,607005	****

Effect	Mauchley Sphericity Test (Dataset_29.01) Sigma-restricted parameterization Effective hypothesis decomposition			
	W	Chi-Sqr.	df	p
DDI	0,426892	8,275786	5	0,141677

Effect	Repeated Measures Analysis of Variance with Effect Sizes and Powers (Dataset_29.01) Sigma-restricted parameterization Effective hypothesis decomposition					
	SS	Degr. of Freedom	MS	F	p	Partial eta-squared
Intercept	25007,07	1	25007,07	908,4621	0,000000	0,988036
Error	302,80	11	27,53			
BMI	0,30	3	0,10	1,1662	0,337402	0,095854
Error	2,80	33	0,08			

Effect	Repeated Measures Analysis of Variance with Effect Sizes and Powers (Dataset_29.01) Sigma-restricted parameterization Effective hypothesis decomposition	
	Non-centrality	Observed power (alpha=0,05)
Intercept	908,4621	1,000000
Error		
BMI	3,4985	0,284165
Error		

Effect	Mauchley Sphericity Test (Dataset_29.01) Sigma-restricted parameterization Effective hypothesis decomposition			
	W	Chi-Sqr.	df	p
BMI	0,117939	20,78207	5	0,000891

Effect	Adjusted Univariate Tests for Repeated Measure: DV_1 (Dataset_29.01)<br>Sigma-restricted parameterization<br>Effective hypothesis decomposition						
	Degr. of Freedom	F	p	G-G Epsilon	G-G Adj. df1	G-G Adj. df2	G-G Adj. p
BMI	3	1,166170	0,337402	0,543038	1,629115	17,92026	0,323981
Error	33						

Effect	Adjusted Univariate Tests for Repeated Measure: DV_1 (Dataset_29.01)<br>Sigma-restricted parameterization<br>Effective hypothesis decomposition						
	H-F Epsilon	H-F Adj. df1	H-F Adj. df2	H-F Adj. p	Lowr.Bnd Epsilon	Lowr.Bnd Adj. df1	Lowr.Bnd Adj. df2
BMI	0,624252	1,872755	20,60031	0,328303	0,333333	1,000000	11,00000
Error							

Effect	Adjusted Univariate Tests for Repeated Measure: DV_1 (Dataset_29.01) Sigma-restricted parameterization Effective hypothesis decomposition
	Lowr.Bnd Adj. p
BMI	0,303298
Error	

Effect	Repeated Measures Analysis of Variance with Effect Sizes and Powers (Dataset_29.01)<br>Sigma-restricted parameterization<br>Effective hypothesis decomposition					
	SS	Degr. of Freedom	MS	F	p	Partial eta-squared
Intercept	242237,9	1	242237,9	389,1546	0,000000	0,972511
Error	6847,2	11	622,5			
WEIGHT	3,2	3	1,1	1,2063	0,322732	0,098827
Error	28,9	33	0,9			

Effect	Repeated Measures Analysis of Variance with Effect Sizes and Powers (Dataset_29.01)<br>Sigma-restricted parameterization<br>Effective hypothesis decomposition	
	Non-centrality	Observed power (alpha=0,05)
Intercept	389,1546	1,000000
Error		
WEIGHT	3,6189	0,293154
Error		

Effect	Mauchley Sphericity Test (Dataset_29.01)<br>Sigma-restricted parameterization<br>Effective hypothesis decomposition			
	W	Chi-Sqr.	df	p
WEIGHT	0,171723	17,12930	5	0,004261

Effect	Adjusted Univariate Tests for Repeated Measure: DV_1 (Dataset_29.01)<br>Sigma-restricted parameterization<br>Effective hypothesis decomposition						
	Degr. of Freedom	F	p	G-G Epsilon	G-G Adj. df1	G-G Adj. df2	G-G Adj. p
WEIGHT	3	1,206317	0,322732	0,541935	1,625804	17,88384	0,313479
Error	33						

Effect	Adjusted Univariate Tests for Repeated Measure: DV_1 (Dataset_29.01)<br>Sigma-restricted parameterization<br>Effective hypothesis decomposition						
	H-F Epsilon	H-F Adj. df1	H-F Adj. df2	H-F Adj. p	Lowr.Bnd Epsilon	Lowr.Bnd Adj. df1	Lowr.Bnd Adj. df2
WEIGHT	0,622618	1,867855	20,54641	0,316927	0,333333	1,000000	11,00000
Error							

Effect	Adjusted Univariate Tests for Repeated Measure: DV_1 (Dataset_29.01) Sigma-restricted parameterization Effective hypothesis decomposition
	Lowr.Bnd Adj. p
WEIGHT	0,295522
Error	

Effect	Repeated Measures Analysis of Variance with Effect Sizes and Powers (Dataset_29.01) Sigma-restricted parameterization Effective hypothesis decomposition					
	SS	Degr. of Freedom	MS	F	p	Partial eta-squared
Intercept	1969,922	1	1969,922	3855,290	0,000000	0,997155
Error	5,621	11	0,511			
HBA1C	0,464	3	0,155	5,189	0,004775	0,320524
Error	0,984	33	0,030			

Effect	Repeated Measures Analysis of Variance with Effect Sizes and Powers (Dataset_29.01) Sigma-restricted parameterization Effective hypothesis decomposition	
	Non-centrality	Observed power (alpha=0,05)
Intercept	3855,290	1,000000
Error		
HBA1C	15,567	0,891695
Error		

Cell No.	Tukey HSD test; variable DV_1 (Dataset_29.01) Homogenous Groups, alpha = ,05000 Error: Within MS = ,02980, df = 33,000			
	HBA1C	DV_1 Mean	1	2
4	V6 HbA1c [%]	6,291667	****	
3	V5 HbA1c [%]	6,358333	****	

Cell No.	Tukey HSD test; variable DV_1 (Dataset_29.01) Homogenous Groups, alpha = ,05000 Error: Within MS = ,02980, df = 33,000			
	HBA1C	DV_1 Mean	1	2
2	V4 HbA1c [%]	6,416667	****	****
1	V3 HbA1c [%]	6,558333		****

Effect	Mauchley Sphericity Test (Dataset_29.01) Sigma-restricted parameterization Effective hypothesis decomposition			
	W	Chi-Sqr.	df	p
HBA1C	0,543306	5,931356	5	0,312956

Effect	Repeated Measures Analysis of Variance with Effect Sizes and Powers (Dataset_29.01) Sigma-restricted parameterization Effective hypothesis decomposition					
	SS	Degr. of Freedom	MS	F	p	Partial eta-squared
Intercept	103878,7	1	103878,7	1701,744	0,000000	0,993578
Error	671,5	11	61,0			
HBA1CM	55,4	3	18,5	5,189	0,004775	0,320524
Error	117,5	33	3,6			

Effect	Repeated Measures Analysis of Variance with Effect Sizes and Powers (Dataset_29.01) Sigma-restricted parameterization Effective hypothesis decomposition	
	Non-centrality	Observed power (alpha=0,05)
Intercept	1701,744	1,000000
Error		
HBA1CM	15,567	0,891695
Error		

Cell No.	Tukey HSD test; variable DV_1 (Dataset_29.01) Homogenous Groups, alpha = ,05000 Error: Within MS = 3,5606, df = 33,000			
	HBA1CM	DV_1 Mean	1	2
4	V6 HbA1c [mmol/l]	45,26792	****	
3	V5 HbA1c [mmol/l]	45,99658	****	
2	V4 HbA1c [mmol/l]	46,63417	****	****
1	V3 HbA1c [mmol/l]	48,18258		****

Effect	Mauchley Sphericity Test (Dataset_29.01)<br>Sigma-restricted parameterization<br>Effective hypothesis decomposition			
	W	Chi-Sqr.	df	p
HBA1CM	0,543306	5,931356	5	0,312956

Effect	Repeated Measures Analysis of Variance with Effect Sizes and Powers (Dataset_29.01)<br>Sigma-restricted parameterization<br>Effective hypothesis decomposition					
	SS	Degr. of Freedom	MS	F	p	Partial eta-squared
Intercept	4466007	1	4466007	2487,453	0,000000	0,995996
Error	17954	10	1795			
FRUCTO	1179	3	393	2,254	0,102467	0,183918
Error	5230	30	174			

Effect	Repeated Measures Analysis of Variance with Effect Sizes and Powers (Dataset_29.01)<br>Sigma-restricted parameterization<br>Effective hypothesis decomposition	
	Non-centrality	Observed power (alpha=0,05)
Intercept	2487,453	1,000000
Error		
FRUCTO	6,761	0,513658
Error		

Effect	Mauchley Sphericity Test (Dataset_29.01)<br>Sigma-restricted parameterization<br>Effective hypothesis decomposition			
	W	Chi-Sqr.	df	p
FRUCTO	0,431745	7,325976	5	0,197504
